# Thermo-mechanical properties prediction of Ni-reinforced Al_2_O_3_ composites using micro-mechanics based representative volume elements

**DOI:** 10.1038/s41598-022-14685-x

**Published:** 2022-06-30

**Authors:** M. M. Shahzamanian, S. S. Akhtar, A. F. M. Arif, W. J. Basirun, K. S. Al-Athel, M. Schneider, N. Shakelly, Abbas Saeed Hakeem, Abba A. Abubakar, P. D. Wu

**Affiliations:** 1 Department of Mechanical Engineering, McMaster University, Hamilton, ON Canada; 2 Department of Mechanical Engineering, King Fahd University of Petroleum & Minerals (KFUPM), Dhahran, Saudi Arabia; 3 Interdisciplinary Research Center for Intelligent Manufacturing and Robotics, KFUPM, Dhahran, Saudi Arabia; 4 McMaster Manufacturing Research Institute (MMRI), Department of Mechanical Engineering, McMaster University, Hamilton, Canada; 5 Department of Chemistry, Universiti Malaya, Kuala Lumpur, Malaysia; 6 Interdisciplinary Research Center for Advanced Materials, KFUPM, Dhahran, 31261 Saudi Arabia; 7 Institute of Engineering Mechanics, Karlsruhe Institute of Technology (KIT), Karlsruhe, Germany; 8 Department of Environmental and Ecological Engineering, Purdue University, West Lafayette, IN USA; 9 Interdisciplinary Research Center for Hydrogen and Energy Storage, KFUPM, Dhahran, 31261 Saudi Arabia

**Keywords:** Materials science, Mathematics and computing, Mechanical engineering

## Abstract

For effective cutting tool inserts that absorb thermal shock at varying temperature gradients, improved thermal conductivity and toughness are required. In addition, parameters such as the coefficient of thermal expansion must be kept within a reasonable range. This work presents a novel material design framework based on a multi-scale modeling approach that proposes nickel (Ni)-reinforced alumina (Al_2_O_3_) composites to tailor the mechanical and thermal properties required for ceramic cutting tools by considering numerous composite parameters. The representative volume elements (RVEs) are generated using the DREAM.3D software program and the output is imported into a commercial finite element software ABAQUS. The RVEs which contain multiple Ni particles with varying porosity and volume fractions are used to predict the effective thermal and mechanical properties using the computational homogenization methods under appropriate boundary conditions (BCs). The RVE framework is validated by the sintering of Al_2_O_3_-Ni composites in various compositions. The predicted numerical results agree well with the measured thermal and structural properties. The properties predicted by the numerical model are comparable with those obtained using the rules of mixtures and SwiftComp, as well as the Fast Fourier Transform (FFT) based computational homogenization method. The results show that the ABAQUS, SwiftComp and FFT results are fairly close to each other. The effects of porosity and Ni volume fraction on the mechanical and thermal properties are also investigated. It is observed that the mechanical properties and thermal conductivities decrease with the porosity, while the thermal expansion remains unaffected. The proposed integrated modeling and empirical approach could facilitate the development of unique Al_2_O_3_-metal composites with the desired thermal and mechanical properties for ceramic cutting inserts.

## Introduction

Al_2_O_3_ based ceramics are currently the most mature ceramic cutting tool material due to their resistance towards thermal shocks, chemical stability, refractory characteristics, and well-established development routes such as sintering. However, its intrinsic brittleness and low thermal conductivity are the major disadvantages for cutting applications. Many attempts have been made to enhance the stiffness, toughness, and thermal conductivity of Al_2_O_3_ without compromising much on its desired low coefficient of thermal expansion which is an important requirement in tool materials particularly for intermittent machining operations. The incorporation of metal particles in Al_2_O_3_ is expected to increase the thermal conductivity and toughness due to the intrinsic thermal and structural properties, hence are potential candidates for Al_2_O_3_-matrix composites. Thus, the implementation of computational homogenization to tailor the Ni-toughened Al_2_O_3_ properties is required for a better design before the fabrication of the composite materials. The properties of heterogeneous materials such as Al_2_O_3_ composites, which are composed of various phases, can be tailored using multiscale modeling (MM). This approach leads to an estimation of the resulting effective thermal and mechanical properties of the composites, at the same time taking into consideration the intrinsic properties of the particles and the matrix material. These properties are then utilized in the microscopic level RVE simulations. The upscaling approach based on RVE(s) permits quantifying the influence of both material and geometric parameters on the effective mechanical properties of the material under consideration^1–4^.

It is imperative to study the effective properties of Al_2_O_3_ since they affect the thermal performance and other properties such as thermal shock resistance, modulus of elasticity, and electrical conductivity^5–8^. The porosity, volume fraction, and distribution have a significant effect on the effective thermal conductivity^9,10^. It is well established that the fracture toughness of brittle Al_2_O_3_ ceramic can be increased through the incorporation of ductile metals^11^. There is a great potential for using ceramic–metal composites in different engineering fields owing to their enhanced thermal, mechanical, and electrical properties. The processing and physical properties of metal/ceramic composites are reported frequently in the literature^12,13^. The interfacial thermal resistance in a composite between different constituent phases is due to a combination of poor chemical and mechanical adherence at the interface and a thermal expansion mismatch of these phases. This interfacial resistance is usually called the Kapitza resistance, named after Kapitza who discovered the presence of discontinuity in the temperature distribution at the metal-liquid interface. The interfacial thermal resistance is reported to be dramatically influenced by the thermal conductivity of different composite materials^14^.

The effective material properties of materials with random (stationary and ergodic) microstructure may be determined through RVE-based homogenization whenever there is sufficient scale separation between the microscopic and the macroscopic scale^1,2,15–19^. Pioneered by Hill^1^, a representative volume element (RVE) is a volume which is statistically entirely typical of the underlying random material and large enough to render the influence of imposed boundary conditions negligible^20–22^. The size of the RVE must be larger than the statistically representative sub-domains of the microscopic geometry to avoid obtaining random results for the effective properties. Willis^2^ used variational and other related methods to calculate the effective thermal and mechanical properties of the fiber-reinforced composites. Qing^16^ generated RVEs for SiC/Al metal matrix composites and analyzed the behavior of a specific metal under tensile, shear and combined tensile/shear loads. Qin et al.^23^ also identified the stress state dependent fracture micro-mechanisms for DP600 steel materials through simulations on the RVEs. Meng et al.^24^ considered the martensitic phase transformation kinetics through the multiscale constitutive models and RVE simulation for metastable metal foils and determined the plastic deformation. Shahzamanian et al.^25^ computed the homogenized mechanical properties in cement paste RVEs and investigated the stress wave propagation in heterogeneous and homogenous RVEs. With regards to the calculated effective mechanical properties, attenuation in the stress wave propagation and shock wave decay were clearly observed in the heterogenous RVE compared to the homogeneous RVE. Benyahi et al.^26^ performed homogenization and calculated the effective material properties for the composite material and determined the damage evolution in an RVE at a micro-scale which eventually leads to failure. Breuer and Stommel^27^ created an artificial neural network that was based on an RVE database to predict the short fiber composite properties. Shen et al.^28^ predicted the thermal and mechanical properties of the SiC_f_/SiC RVE using finite element method (FEM) and asymptotic homogenization approach with detailed presentation and implementation of the process. Kaminski et al.^29^ calculated the heat transfer using a homogenization technique in fibrous composite with stochastic interface defects. Furthermore, the stress–strain curve determination, as well as the fracture analysis in various materials could be obtained through the simulation on the RVEs^18,19,30–35^.

For predicting the effective properties of the composites, various homogenization techniques are available^36–41^. Hill^1^ in 1963 presented some theoretical principles for the calculation of the elastic properties of reinforced solids with perfectly bonded two isotropic phases. The energy approach described by Hill^1^ involves the average stress and strain for the calculation of the effective elastic properties. Hashin and Shtrikman^15^ in 1963 proposed variational principles to derive upper and lower bounds for the elastic properties of multiphase materials. The obtained results were in good agreement with the experimental data especially for small moduli contrast between the phases. The computing methodology of the mechanical properties of a heterogeneous material containing various phases (cement paste) was presented previously^42,43^. For this purpose, the appropriate BCs are applied on the RVE by using the commercial finite element ABAQUS software. The boundary conditions, such as the homogeneous boundary conditions (HBC) and the periodic boundary conditions (PBC) should be imposed on the RVE in such a way that the deformed shape of a homogeneous RVE remains cubic to avoid emerging stress concentration. It should be noted that the PBC is imposed to the RVEs to eliminate the edge effects in the RVEs for the prediction of effective material properties. The kinematic uniform boundary condition (KUBC) and stress uniform boundary conditions (SUBC) are the two types of HBCs^44^. In KUBC, displacements are applied to the RVE, compared to stresses which are imposed on the RVE in SUBC. It was found^45^ that the results of elastic properties for PBC and KUBC are close to each other but higher than the results for SUBC. As stated by Kanit et al.^46,47^, the value difference between the KUBC and SUBC decreases with increase in the volume element. Moreover, the apparent Young’s modulus of an RVE possesses the highest value when constrained at the periphery surfaces. To calculate the thermal conductivity of the RVEs, the heat fluxes are initially exerted on two opposite surfaces of the RVE and the temperature variation is then computed. The thermal conductivity is obtained from the Fourier’s law^48–50^. Also, a uniform temperature is applied to the RVE and the average volumetric strain is computed to investigate the effective thermal expansion coefficient. This value is obtained by dividing the average volumetric strain by the applied temperature value^51^.

Different tools and software can be used to calculate the effective properties of RVEs for the composite materials. Before using the finite element software such as ABAQUS, the appropriate boundary conditions must be imposed on the RVEs. However, the presence of interfaces allows to compute the effective properties without imposing any boundary conditions, such as the variational asymptotic method for the unit cell homogenization, (VAMUCH)^52–58^ and SwiftComp, respectively^59–62^. VAMUCH is used as a micromechanics tool for presenting the effective properties of the heterogeneous materials. VAMUCH is a general-purpose micromechanics framework which uses an asymptotic analysis of the variational problem, thereby synthesizing the merits of both variational and asymptotic methods. SwiftComp may also be used in conjunction with a commercial finite element software to model composites and perform a structural analysis and design through the implementation of mechanics of a structure genome (MSG)^63^. All the constitutive information for the structure genome (SG) must be identified to be homogenized by the SwiftComp^64^. A SG can be simulated as a one-dimensional (1D), two-dimensional (2D) and three-dimensional (3D) model depending on the degree of heterogeneity of a heterogeneous material^60^. Another method which has been frequently used in recent years is the computational homogenization methods based on the Fast Fourier Transform (FFT)^65,66^. The homogenized properties in the RVE are calculated based on the discretization of regular grid in terms of the identical brick-shaped elements. Therefore, this approach is ideally suitable for digital-volume images. This method has been recognized as an effective alternative to the classical finite-element based homogenization techniques, as it rapidly predicts the effective properties with minimum computational effort^67^.

The basic theme of this work is to use various computational schemes validated through novel Spark plasma sintering (SPS) synthesis route for the ceramic composites intended for cutting inserts with the tailorable thermal and structural applications. The goal is to predict the best combination of the Al_2_O_3_ matrix with Ni particles in terms of Ni attributes, volume loading, and porosity. The primary focus is to predict the homogenized properties at macro levels using the micromechanics-based models. This study focuses on the prediction of the thermo-mechanical properties of Al_2_O_3_-Ni when small strain quasi-static loading conditions are imposed on the microscale domains. The RVEs for the Al_2_O_3_ material containing Ni particles with various volume fractions and porosities are generated by Dream.3D and then imported into the ABAQUS software. Elements with very low thermal and mechanical properties (almost zero) are distributed within the RVEs to investigate the effects of physical porosities on the thermal and mechanical properties directly. The physical porosities are generated in the RVE with low thermal and mechanical properties. The thermal and mechanical properties such as the Young’s, shear and bulk moduli, as well as the thermal expansion and thermal conductivity are computed by imposing the appropriate boundary conditions. Firstly, the isotropy of the RVEs is investigated by applying a displacement in each of the coordinate directions. The results are generated by selecting an appropriate size of the considered volume element which encompasses a sufficient number of particles to reproduce the statistics/characteristics the real material. The effective properties are computed using other methods such as the rules of mixture, SwiftComp, and FFT-based computational homogenization. A brief comparison result is performed to investigate the accuracy of each method. The proposed model is validated by developing composite systems of the Al_2_O_3_ matrix and Ni particles. Spark plasma sintering (SPS) process is used for synthesis of composites, which is considered a novel sintering method. The effect of this sintering technique has a direct bearing on the properties and hence a direct relationship with the calibration of the current modeling approaches. Lastly, it must be emphasized that although FFT and SwiftComp methods rapidly provide the effective properties with minimum computational effort, the aim of results comparison with other techniques is to verify the accuracy of the RVE-based model in a finite element software ABAQUS. This study paves the way for further investigations of Al_2_O_3_ matrix containing Ni particles such as brittle fracture analysis etc. using the RVE-based model in ABAQUS.

## Experimental methodology

In this section, the experimental methodology including the materials analyzed in this study and the sample preparations are presented. In this regard, the experimental methodology will be discussed in detail.

### Materials and Synthesis of Samples

Ni powders were incorporated into the matrix material which is Al_2_O_3_ to fabricate the composite. The α-Al_2_O_3_ powder with an average particle size of 0.8 µm and nickel particles with an average size of 90 µm were supplied by BUEHLER and SANDVIK OSPREY, respectively. Table 1^68,69^ shows the thermal and mechanical properties of the pure Al_2_O_3_ and Ni particles used in this study. The homogenized properties of the composite samples were then measured and compared well with those obtained using prediction models for the sake of validation.

**Table 1:** Properties of Ni particles and monolithic Al_2_O_3_^68,69^.

Property	Ni	Pure sintered Al_2_O_3_
Elastic modulus, GPa	200	332
Poisson’s ratio	0.31	0.22
Density, g/cm^3^	8.89	3.99 (measured experimentally)
Thermal conductivity, W/m.K	90.9	24.5 (measured experimentally)
Coefficient of thermal expansion, 1/°C	13.4 × 10^–6^	7.1 × 10^–6^ (measured experimentally)

Firstly, a mechanical planetary ball milling apparatus was used to disperse the Ni particles into the Al_2_O_3_ matrix at a sufficiently low speed of 150 rpm for 90 min before sintering. Then, in order to mix the two powders, the ball milling apparatus was carefully operated without the milling balls to avoid crushing and/or decreasing the particles size. An ultrasonic probe sonicator (Model VC 750, SONICS, USA) was used to homogenize the composite samples, and ethanol was used as the homogenizing medium. Later, the samples were placed in a furnace at 80 °C for 24 h to evaporate the ethanol. The Spark Plasma Sintering (SPS) technique was used to sinter the Al_2_O_3_–Ni composites containing 5%, 10%, 15%, and 20% Ni particles. A 30 mm diameter graphite die was used to develop the composite samples using an automatic SPS equipment from FCT, (Germany). It is important to realize that the SPS parameters have a significant impact on the quality of composites, hence preliminary testing was carried out to optimize the composites process. The sintering temperature was varied between 1200 °C and 1400 °C, with a holding time of 10 to 20 min and a pressure of 35 to 50 MPa. Sintering at 1400 °C for 10 min with a pressure of 50 MPa produces the best densification results, which are taken into account in all experiments. The sintering was performed at a heating rate of 100 °C/min. For a full explanation and operational procedures of the equipment, we refer to Akhtar et al.^70^.

### Testing and characterization techniques

The sample preparation for the microscopy was performed by a JEOL JSM-6460LV (Japan) Field Emission Scanning Electron Microscope (FESEM). The specimens were cut to cross sections, found polished, and coated with a thin film of gold by vacuum evaporation to improve the light penetration leading to better surface micrographs and higher quality. The composites were characterized for possible the formation of phases X-ray diffraction analysis for performed on a RIGAKU desktop x-ray diffractometer model "MINIFLEX II" with copper radiation and a wavelength of 1.5418A. The thermal conductivities of the developed composites at room temperature were measured using the C-Therm TCI Thermal Conductivity Analyzer. The Modified Transient Plane Source (MTPS) equipment was used in the thermal conductivity experiments. The thermal conductivity of the samples was measured directly because the MTPS technique uses a one-sided interfacial heat reflectance sensor, and it applies a momentary constant heat source to the samples. A METTLE TOLEDO thermal mechanical analyzer (TMA/SDTA 1 LF/1100, USA) was used to measure the coefficient of thermal expansion of the composites. Very small samples with dimensions of 3 mm × 3 mm × 2 mm were cut by a diamond cutter to place the samples in the equipment. The Archimedes principle or water displacement method (ASTM D792-91) was applied to calculate the actual or measured density (${\rho }_{ac}$) of the composites experimentally. The rule of mixtures was employed to determine the theoretical density (${\rho }_{th}$) of the composite based on the density and volume fraction of the matrix and particles.

1 $$\begin{gathered} \rho_{th} = \rho_{mat} \phi_{mat} + \rho_{inc} \phi_{inc} \hfill \\ \phi_{mat} + \phi_{inc} = 1 \hfill \\ \end{gathered}$$

where *ρ* and *ϕ* denote the density and volume fractions, respectively. The subscripts *mat* and *inc* represent the matrix and inclusion materials, respectively. Furthermore, the percentage porosity ($\%P$) or the volume fraction of voids (${v}_{v}$) of the composites were determined through the following formula.

2 $$\begin{aligned} \% P & = \left( {\frac{{\rho_{th} - \rho_{ac} }}{{\rho_{th} }}} \right) \times 100 \\ v_{v} & = \left( {\frac{{\rho_{th} - \rho_{ac} }}{{\rho_{th} }}} \right) \\ \end{aligned}$$

The elasticity modulus was measured by a MICRO COMBI TESTER from CSM Instruments (USA) through indentation by a pyramid diamond onto the surface of the samples. First, the indenter was loaded to a preset value, then it was unloaded gradually until material relaxation occurs. The following equation based on the slope of the tangent to the loading curve, is used to calculate the elastic modulus of the samples:

3 $${E}_{IT}=\frac{1-{{v}_{s}}^{2}}{\frac{1}{{E}_{r}}-\frac{{{v}_{i}}^{2}}{{E}_{i}}}$$

where *ν*_*s*_ is the Poisson’s ratio of the sample, *E*_*i*_ and *ν*_*i*_ are the elastic modulus and Poisson’s ratio of the diamond indenter which are 1141GPa and 0.07, respectively. The reduced modulus (*E*_*r*_*)* is obtained using the data from the indentation as follows:

4 $${E}_{r}=\frac{\sqrt{\pi }\cdot S}{2\cdot b\cdot \sqrt{{A}_{P}({h}_{c})}}$$

In Eq. (4), S is the slope of the unloading curve, b is the compliance constant, *h*_*c*_ is the contact depth and *A*_*p*_ is the projected contact area.

## Experimental results and discussion

**Figure 1:**
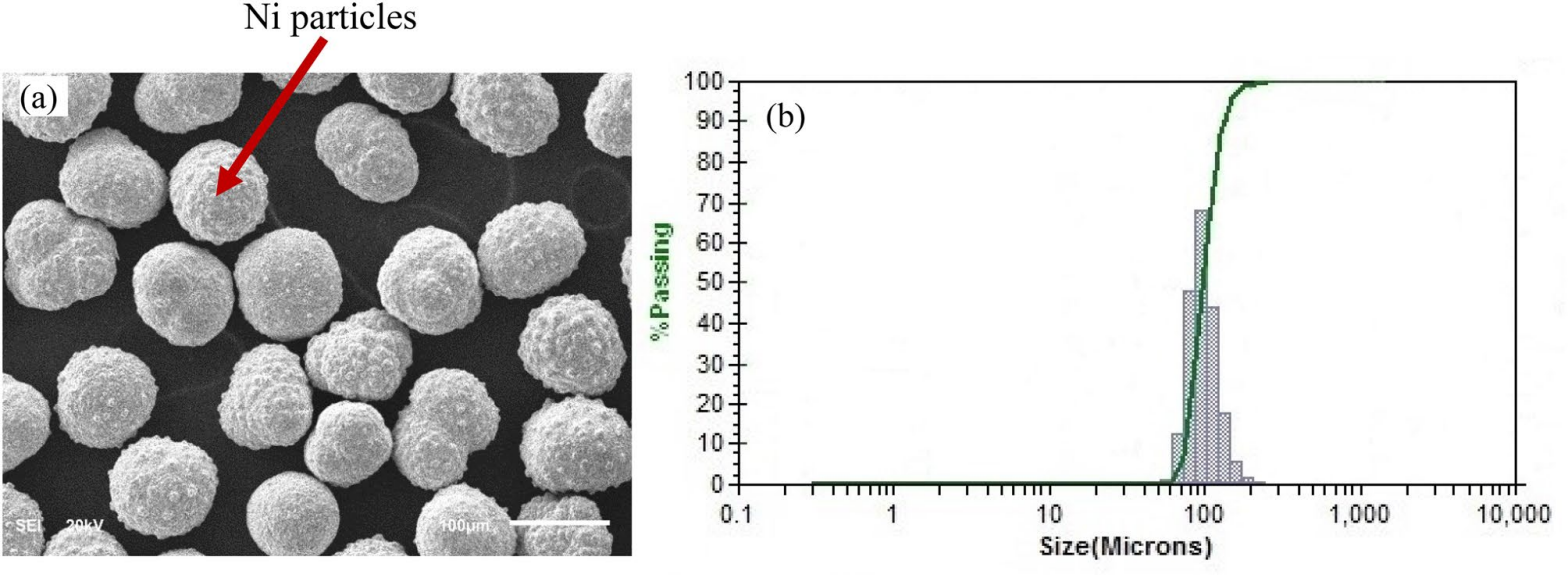
(**a**) FESEM image of Ni particles used in the sintering of Ni-reinforced Al_2_O_3_ composites. The Ni particles are nearly spherical, (**b**) The Ni particle size analysis shows a normal distribution with a mean particle size between 90 and 100 µm.

Figure 1 shows the FESEM images of Ni particles used in the current work. The particle size distribution was investigated using the particle size analysis. The average particle size is in the range of 90 µm to 100 µm. We observed that the particles are roughly equiaxed, which is also assumed in the RVE-model by assuming a spherical shape of the particles for validation purposes.

**Figure 2:**
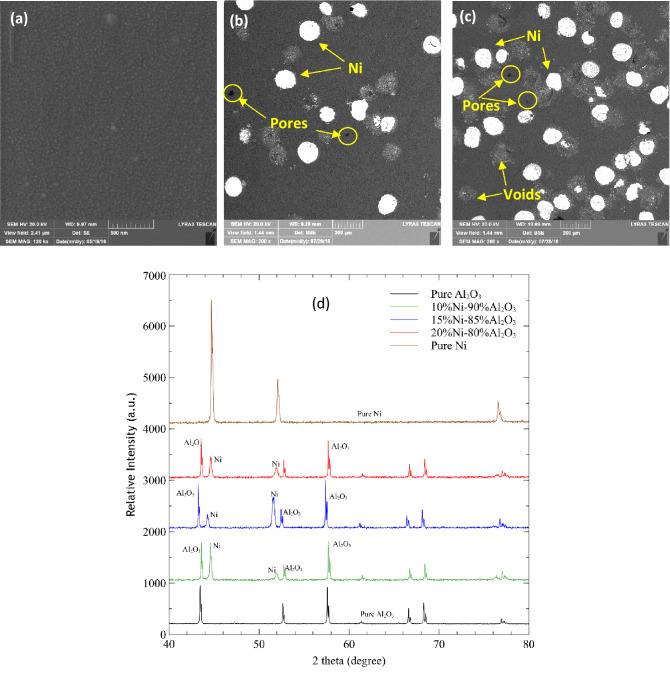
FESEM images of sintered pure Al_2_O_3_ and Ni-reinforced Al_2_O_3_ composites prepared using spark plasma sintering process. (**a**) Pure Al_2_O_3_, (**b**) Al_2_O_3_-10%Ni composites, and (**c**) Al_2_O_3_-20%Ni composite. The porosity increases with the percentage of Ni. Some voids are also visible which were behind after ejection of Ni particles during polishing of samples. (**d**) XRD spectra of synthesized Ni-reinforced Al_2_O_3_ composites with 10%, 15%, and 20% Ni content. The XRD peaks of pure Ni and pure Al_2_O_3_ are also shown.

Figure 2 shows representative micrographs of pure Al_2_O_3_, and its composites with 10% and 20% vol. Ni, respectively. All SEM images show a homogenous distribution of particles, which indicates the effective processing route adapted in this work. In these images, the Ni particles appear brighter compared to the Al_2_O_3_ matrix. The porosity in the resulting composites is strongly affected by the volume loading when the samples are sintered under homogenous synthesis conditions. Table 2 shows the porosity of the Al_2_O_3_–Ni composites as a function of Ni loading.

**Table 2:** Measured porosity volume fraction for Al_2_O_3_ matrix composites.

Ni Volume Fraction (%) in Al_2_O_3_ matrix composites	Porosity (%)
0	0.25
5	0.24
10	0.90
15	1.27
20	4.22

It is also observed that the level of porosity is linearly dependent on the percentage of Ni content in the Al_2_O_3_ matrix with almost no porosity in the case of pure Al_2_O_3_ while a maximum level is achieved in the 20% Ni composite. The sintering temperature used in the synthesis was slightly less than the melting point of Ni and hence the melting of particles may not occur. However, some Ni was found to form a layer on the surfaces of the synthesized samples, indicating that some of the Ni particles melted and escaped from the graphite die during the SPS at 1400 °C. Similar findings have been reported earlier^71,72^. This phenomenon occurred only from the outer periphery of the samples which is due to the contact of punch and powder at high pressure. It is worth mentioning that the ejection of the Ni was very slight and not significant that could lead to inhomogeneous structure of the composites at the core as evident from SEM images. As the nickel content increased, so did the amount of nickel that melted, resulting in comparatively lower relative densities. Although no clear explanation for the increase in the porosity has been found in the literature, another possibility is that it is related to mismatch of the coefficient of thermal expansion of Al_2_O_3_ and Ni, which results in the formation of void spaces at the Al_2_O_3_/Ni interface^72^. As can be seen in SEM images, the Ni particles are distributed evenly throughout the Al_2_O_3_ matrix and hence no inhomogeneous microstructure is detected. The composites were found to be well consolidated as evident form microstructure. To confirm if there is any chemical reaction occurred between the Al2O3 and Ni, XRD analysis performed (Fig. 2d). X–ray diffraction analysis further reveals that the composites are primarily composed of alumina and nickel and no reaction had occurred between them.

Some voids are also observed in the Al_2_O_3_-Ni composites in addition to porosity as indicated in Fig. 2c, which should not be confused with the porosity. These voids arise from the peeling-off of the Ni particles during the sample preparation for microscopy, which indicates that the Ni particles could influence the mechanical properties, such as the resulting toughness due to the incorporation of ductile particles into the Al_2_O_3_ matrix.

## Generation of representative volume elements

**Figure 3:**
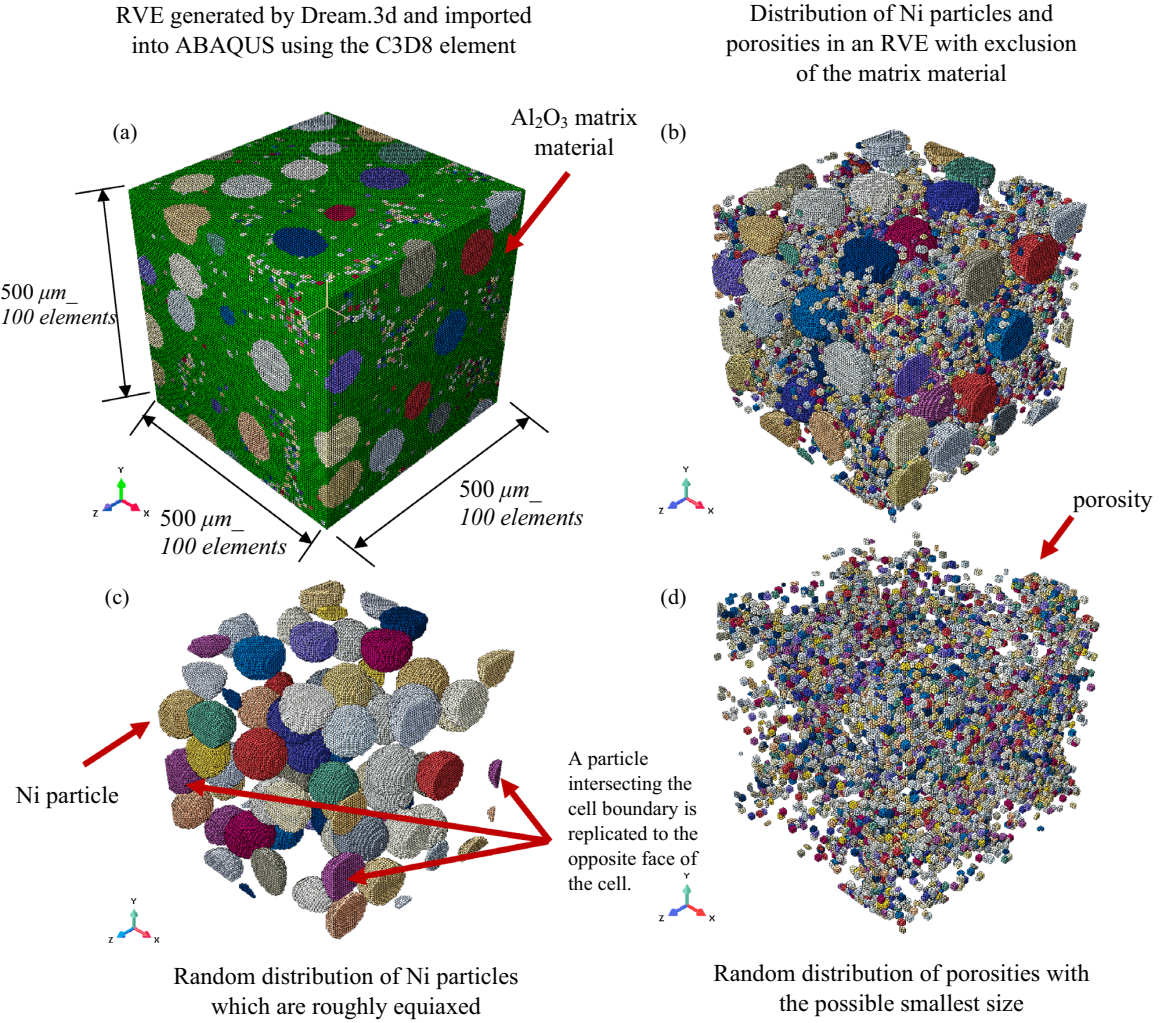
Isometric view of (**a**) a typical Al_2_O_3_ RVE containing 20% Ni particles and 4.22% porosities created by DREAM.3D imported into finite element software ABAQUS for the calculation of the mechanical and thermal properties by the imposition of appropriate boundary conditions at 500 μm length containing 100 elements in each direction; (**b**) Ni particles and porosity distributions when the matrix material is excluded; (**c**) Ni particles distribution which are roughly equiaxed and the experimentally calculated particles size distribution inserted into Dream.3d. A particle intersection of the cell boundary is replicated to the opposite face of the cell; and (**d**) porosities distributions in the RVE when the possible smallest particle size distribution is inserted into the Dream.3d. The Ni particles and porosities are distributed randomly in the RVE.

In this section, the generation of an RVE through a voxel-based finite element approach is described when the required statistical data calculated in the experiments were inserted into Dream. 3D and the output were imported into ABAQUS. The RVEs containing various Ni particles and porosity volume fractions were generated using the Dream.3D software^73^. The required data inserted into Dream.3D to generate the particles are the size, shape, spherical distribution and the radial distribution function (RDF) of the particles. For the size distribution, $\mu =4.55$ and $\sigma =0.02$ are selected for the Ni particles with nearly 100 μm average diameter. The particles are considered to be fully spherical and the automatic RDF generation by the Dream.3D is adopted. We use the same procedure to distribute the porosities in the RVEs, where the parameters $\mu =2.3$ and $\sigma =0.1$ were used to form the porosities with the smallest volume generated by the software. Dream.3D produces fully microstructures, i.e., a particle intersecting the cell boundary is copied to the opposite face of the cell. The Al_2_O_3_ matrix material is considered homogeneous without any grain distribution. The RVEs generated were imported into a commercial finite element software ABAQUS. Figure 3 shows the generated RVE containing 20% Ni particles and 4.22% porosities with a 500 μm length containing 1 million (1 M) elements. In this study, the continuum hexahedral 8-node linear brick (C3D8) element and the 8-node linear heat transfer brick (DC3D) were used for the mechanical and thermal analysis, respectively.

Although tetrahedral elements are more suitable than hexahedral elements in a 3D RVE, Dream.3D can only provide hexahedral elements in ABAQUS^74^. However, so many 3D RVEs containing particles were created using the hexahedral elements such as those for cement paste materials created by CEMHYD3D programs. All these RVEs were successful to predict the effective material properties under various boundary conditions^75–77^.

## Properties prediction using homogenization

In this section, the methods are explained in detail, such as the homogenization techniques in ABAQUS by applying appropriate boundary conditions, traditional rules of mixtures, SwiftComp, and FFT to predict the mechanical and thermal properties.

### Microstructure based homogenization using ABAQUS

**Figure 4:**
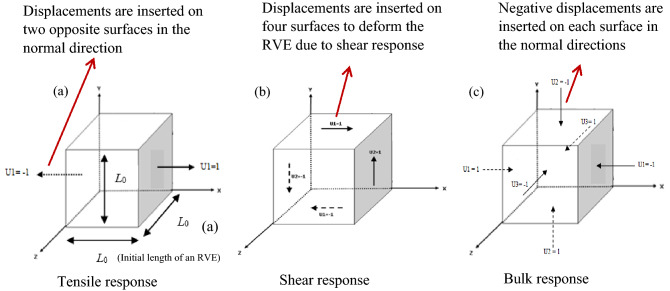
Prescribed kinematic uniform boundary condition (KUBC): (**a**) tensile deformation when the displacements are imposed on two opposite surfaces in the tensile direction; (**b**) shear deformation when the displacements are imposed on the four surfaces to deform the RVE due to shear; and (**c**) bulk deformation when the negative displacements are imposed on each surface of the RVE^45^.

In this sub-section, the appropriate boundary conditions imposed on an RVE to predict the mechanical and thermal properties are explained in detail. To compute the homogenized mechanical properties such as the Young’s, shear and bulk moduli, the kinematic uniform boundary condition (KUBC) and periodic boundary condition (PBC) were imposed on the RVEs. For the KUBC, the uniform displacement is imposed on the face with *x* = *L*_0,_ and a displacement is imposed on the face with *x* = 0 in the opposite direction (Fig. 4a) for tensile loading. For the pure shear loading, uniform displacements are imposed on all the nodes of faces with *x* = *L*_0,_ and *y* = *L*_0_. Displacements are also imposed on all nodes of the faces with *x* = 0 and *y* = 0 in opposite directions (Fig. 4b). With regards to the bulk response (Fig. c), a uniform negative displacement is imposed on every node of each face of the RVE.

**Figure 5:**
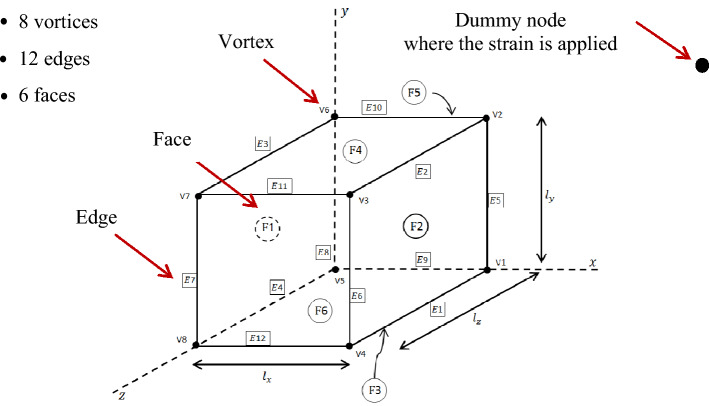
Prescribed periodic boundary conditions (PBC). The vertices, edges and faces are grouped and their displacements are considered in the PBC formula to calculate the effective properties^42^ to avoid the edge effects. The strain is applied on a dummy node and the RVE response is based on the coupled equations constraints. The effective moduli of RVEs is calculated using Eq. (5) based on the energy given by ABAQUS corresponding the overall response of an RVE for a specific applied displacement. The periodicity is imposed on the RVE where every node on the two parallel surfaces responds in a way to maintain the displacment differences of $L\times \varepsilon$.

The periodic boundary conditions (PBCs) are applied to the RVE in order to ensure that the bulk response of the material is simulated without any edge effects (Fig. 5).

As shown below, the equations are formulated such that the differences in displacements between any two opposite faces of the domain are proportional to the applied strain on a dummy node^42,78^.

**Table:** 

Nodes on faces	Nodes on edges	Nodes on vertices
${\mathrm{u}}_{i}^{F2}-{\mathrm{u}}_{i}^{F1}-{L}_{x}{\varepsilon }_{i1}=0$ ${\mathrm{u}}_{i}^{F4}-{\mathrm{u}}_{i}^{F3}-{L}_{y}{\varepsilon }_{i2}=0$ ${\mathrm{u}}_{i}^{F6}-{\mathrm{u}}_{i}^{F5}-{L}_{z}{\varepsilon }_{i3}=0$	${\mathrm{u}}_{i}^{E2}-{\mathrm{u}}_{i}^{E4}-{L}_{x}{\varepsilon }_{i1}-{L}_{y}{\varepsilon }_{i2}=0$ ${\mathrm{u}}_{i}^{E1}-{\mathrm{u}}_{i}^{E3}-{L}_{x}{\varepsilon }_{i1}+{L}_{y}{\varepsilon }_{i2}=0$ ${\mathrm{u}}_{i}^{E6}-{\mathrm{u}}_{i}^{E8}-{L}_{x}{\varepsilon }_{i1}-{L}_{z}{\varepsilon }_{i3}=0$ ${\mathrm{u}}_{i}^{E5}-{\mathrm{u}}_{i}^{E7}-{L}_{x}{\varepsilon }_{i1}+{L}_{z}{\varepsilon }_{i3}=0$ ${\mathrm{u}}_{i}^{E11}-{\mathrm{u}}_{i}^{E9}-{L}_{y}{\varepsilon }_{i2}-{L}_{z}{\varepsilon }_{i3}=0$ ${\mathrm{u}}_{i}^{E10}-{\mathrm{u}}_{i}^{E12}-{L}_{y}{\varepsilon }_{i2}+{L}_{z}{\varepsilon }_{i3}=0$	${\mathrm{u}}_{i}^{V3}-{\mathrm{u}}_{i}^{V5}-{L}_{x}{\varepsilon }_{i1}-{L}_{y}{\varepsilon }_{i2}-{L}_{z}{\varepsilon }_{i3}=0$ ${\mathrm{u}}_{i}^{V2}-{\mathrm{u}}_{i}^{V8}-{L}_{x}{\varepsilon }_{i1}-{L}_{y}{\varepsilon }_{i2}+{L}_{z}{\varepsilon }_{i3}=0$ ${\mathrm{u}}_{i}^{V7}-{\mathrm{u}}_{i}^{V1}+{L}_{x}{\varepsilon }_{i1}-{L}_{y}{\varepsilon }_{i2}-{L}_{z}{\varepsilon }_{i3}=0$ ${\mathrm{u}}_{i}^{V4}-{\mathrm{u}}_{i}^{V6}-{L}_{x}{\varepsilon }_{i1}+{L}_{y}{\varepsilon }_{i2}-{L}_{z}{\varepsilon }_{i3}=0$

The PBCs equations are explained in Ref.^42^ in detail and the procedure is explained carefully. The method to group the nodes at the vertexes ($${v}_{i})$$, edges $${(E}_{i})$$ and surfaces $$({F}_{i})$$ are also described in Ref.^42^ accordingly.

The following equations are used to compute the homogenized Young’s, shear, and bulk moduli:

5 $${E}_{ij}=\frac{2 U}{{(\varepsilon }_{ij}{\varepsilon }_{ij}){V}_{RVE}}{\delta }_{ij}, {G}_{ij}=\frac{2 U}{{(\varepsilon }_{ij}{\varepsilon }_{ij}){V}_{RVE}}\left(1-{\delta }_{ij}\right)\,{\rm and}\,\,K=\frac{2U}{{\varepsilon }_{v}{\varepsilon }_{v}{V}_{RVE}}$$

with the absence of summation on the *i* and *j.* U, ${\varepsilon }_{ij}^{avg},$ and ${V}_{RVE}$ refer to the strain energy of the RVE, the average strain and volume of the RVE, respectively.

The compliance matrix ${(S}_{ij})$ in Voigt notation for a orthotropic material is given by^79^:

6 $$\left\{\begin{array}{l}\begin{array}{l}{\varepsilon }_{11}\\ {\varepsilon }_{22}\\ {\varepsilon }_{33}\end{array}\\ \begin{array}{l}{{\gamma }_{23}=2\varepsilon }_{23}\\ {{\gamma }_{31}=2\varepsilon }_{31}\\ {\gamma }_{12}={2\varepsilon }_{12}\end{array}\end{array}\right\}=\left[\begin{array}{ll}\begin{array}{lll}\frac{1}{{E}_{1}}& -\frac{{\nu }_{21}}{{E}_{2}}& -\frac{{\nu }_{31}}{{E}_{3}}\\ -\frac{{\nu }_{12}}{{E}_{1}}& \frac{1}{{E}_{2}}& -\frac{{\nu }_{32}}{{E}_{3}}\\ -\frac{{\nu }_{13}}{{E}_{1}}& -\frac{{\nu }_{23}}{{E}_{2}}& \frac{1}{{E}_{3}}\end{array}& \begin{array}{lll}0& 0& 0\\ 0& 0& 0\\ 0& 0& 0\end{array}\\ \begin{array}{lll}0& 0& 0\\ 0& 0& 0\\ 0& 0& 0\end{array}& \begin{array}{lll}\frac{1}{{G}_{23}}& 0& 0\\ 0& \frac{1}{{G}_{31}}& 0\\ 0& 0& \frac{1}{{G}_{12}}\end{array}\end{array}\right]\left\{\begin{array}{l}\begin{array}{l}{\sigma }_{11}\\ {\sigma }_{22}\\ {\sigma }_{33}\end{array}\\ \begin{array}{l}{\tau }_{23}\\ {\tau }_{31}\\ {\tau }_{12}\end{array}\end{array}\right\}$$

The following equation shows the relationship between the compliance matrix ${(S}_{ij})$ and stiffness matrix ${(C}_{ij})$.

7 $$\left[C\right]={\left[S\right]}^{-1}$$

The stiffness matrix ${(C}_{ij})$ in Voigt notation for a orthotropic material is given by^79^:

8 $$\left\{\begin{array}{l}\begin{array}{l}{\sigma }_{11}\\ {\sigma }_{22}\\ {\sigma }_{33}\end{array}\\ \begin{array}{l}{\tau }_{23}\\ {\tau }_{31}\\ {\tau }_{12}\end{array}\end{array}\right\}=\left[\begin{array}{ll}\begin{array}{lll}\frac{1-{\nu }_{23}{\nu }_{32}}{{E}_{2}{E}_{3}\Delta }& \frac{{\nu }_{21}+{\nu }_{31}{\nu }_{23}}{{E}_{2}{E}_{3}\Delta }& \frac{{\nu }_{31}+{\nu }_{21}{\nu }_{32}}{{E}_{2}{E}_{3}\Delta }\\ \frac{{\nu }_{12}+{\nu }_{13}{\nu }_{32}}{{E}_{3}{E}_{1}\Delta }& \frac{1-{\nu }_{31}{\nu }_{13}}{{E}_{3}{E}_{1}\Delta }& \frac{{\nu }_{32}+{\nu }_{31}{\nu }_{12}}{{E}_{3}{E}_{1}\Delta }\\ \frac{{\nu }_{13}+{\nu }_{12}{\nu }_{23}}{{E}_{1}{E}_{2}\Delta }& \frac{{\nu }_{23}+{\nu }_{13}{\nu }_{21}}{{E}_{1}{E}_{2}\Delta }& \frac{1-{\nu }_{12}{\nu }_{21}}{{E}_{1}{E}_{2}\Delta }\end{array}& \begin{array}{lll}0& 0& 0\\ 0& 0& 0\\ 0& 0& 0\end{array}\\ \begin{array}{lll}0& 0& 0\\ 0& 0& 0\\ 0& 0& 0\end{array}& \begin{array}{lll}{G}_{23}& 0& 0\\ 0& {G}_{31}& 0\\ 0& 0& {G}_{12}\end{array}\end{array}\right]\left\{\begin{array}{l}\begin{array}{l}{\varepsilon }_{11}\\ {\varepsilon }_{22}\\ {\varepsilon }_{33}\end{array}\\ \begin{array}{l}{{\gamma }_{23}=2\varepsilon }_{23}\\ {{\gamma }_{31}=2\varepsilon }_{31}\\ {\gamma }_{12}={2\varepsilon }_{12}\end{array}\end{array}\right\}$$

$$\Delta = \frac{{1 - \nu_{12} \nu_{21} - \nu_{23} \nu_{32} - \nu_{31} \nu_{13} - 2\nu_{12} \nu_{23} \nu_{31} }}{{E_{1} E_{2} E_{3} }}$$

**Figure 6:**
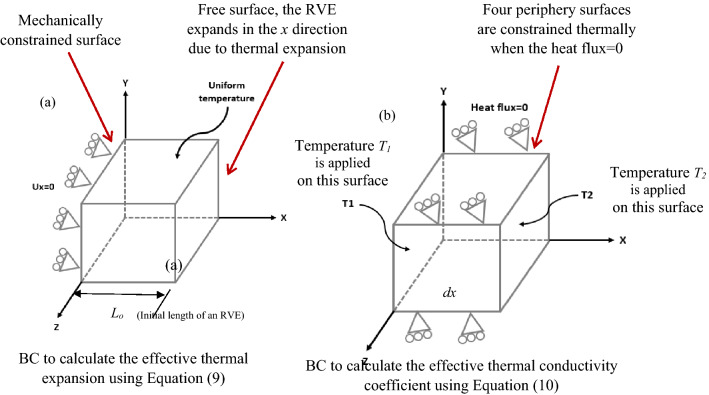
Prescribed thermal boundary conditions imposed on an RVE for the calculation of the; (**a**) effective thermal expansion coefficient when the surface at x = 0 is constrained, and uniform temperature is applied to the RVE. The effective thermal expansion is calculated by obtaining the expansion in the *x* direction given by ABAQUS and inserting into Eq. (9); (**b**) effective thermal conductivity coefficients when the heat flux at the four periphery surfaces becomes zero and uniform different temperatures are applied to the opposite surfaces. The effective thermal conductivity coefficient is calculated by obtaining the heat flux (*q*) given by ABAQUS and inserting into Eq. (10).

In order to calculate the effective thermal expansion coefficient, the nodes located on a single surface of the RVE are constrained and a uniform temperature is applied on the whole RVE as shown in Fig. 6a. The length change ($dL$) of the RVE was computed in the direction where the RVE is constrained and the thermal expansion was calculated by the following equation:

9 $$\alpha = \frac{dL}{{dT}}\frac{1}{{L_{o} }}$$

here, $L_{o}$ is the initial length of the RVE and $dT$ is the applied temperature on the RVE assuming that the initial temperature is zero.

In order to compute the effective thermal conductivity coefficient, uniform temperatures with different values are imposed on two parallel surfaces of the RVE and the periphery boundaries are thermally constrained where the heat fluxes are zero (Fig. 6b). The heat flux $\left( q \right)$ was computed on one side of the RVE where a uniform temperature was applied, and the thermal conductivity coefficient was calculated from Fourier’s law;

10 $$k = - \frac{q}{dT/dx}$$

where $dT$ is the temperature difference on the surface of the RVE and $dx$ is the length of the RVE.

### Rule of mixtures based homogenization

In this sub-section, brief descriptions about the rules of mixtures for the calculation of the homogenized Young’s, shear, and bulk moduli of composites are presented. The aims of the homogenization methods such as the analytical and numerical methods are to determine the effective material properties. However, the analytical methods in contrast to the numerical methods, are established based on simplified assumptions related to the inclusion geometry, boundary conditions or isotropy^80^. In this study, few analytical methods are used to estimate the effective properties of the RVEs and compared to those obtained using numerical results.

A rule of mixture approach which is independent of the microstructure of the material is used to compute the effective bulk properties of the composite materials. The theoretical extreme upper and lower boundaries on the effective material properties of the multi-phase materials are the Voigt^81^ and Reuss^82^ bounds. The rule of the mixture-based Voigt upper bound on the effective bulk (K*) and shear moduli (G*) of a mixture of *n* material phases is given by:

11 $$K^{*} = \mathop \sum \limits_{i = 1}^{n} f_{i} K_{i} \;{\text{and}}\;G^{*} = \mathop \sum \limits_{i = 1}^{n} f_{i} G_{i}$$

The inverse rule of mixture based on the Reuss lower bound on the effective bulk (K*) and shear moduli (G*), is given by:

12 $$\frac{1}{{K^{*} }} = \mathop \sum \limits_{i = 1}^{n} \frac{{f_{i} }}{{K_{i} }}\;{\text{and}}\;\frac{1}{{G^{*} }} = \mathop \sum \limits_{i = 1}^{n} \frac{{f_{i} }}{{G_{i} }}$$

The Hill’s criterion rule of a mixture is based on the average values predicted by the Voigt and Reuss rules^42^.

Hashin^83^ in 1962 presented the composite (or coated) spheres model for determining the effective material properties of multi-phase materials, based on the dilute suspension model. Here, a large number of uniformly distributed and coated spherical inclusions that occupy all the spaces in a matrix are considered. The effective bulk modulus (K*) is given by:

13 $$\frac{{K^{*} }}{{K_{m} }} = 1 + 3\left( {1 - \nu_{m} } \right)\mathop \sum \limits_{i = 1}^{n} \frac{{\left( {\frac{{K_{p}^{i} }}{{K_{m} }} - 1} \right)c_{i} }}{{2\left( {1 - 2\nu_{m} } \right) + \left( {1 + \nu_{m} } \right)\left[ {\frac{{K_{p}^{i} }}{{K_{m} }} - \left( {\frac{{K_{p}^{i} }}{{K_{m} }} - 1} \right)c} \right]}}$$

where, $K_{p}^{i}$, $K_{m}$, and $K^{*}$ are the bulk modulus of the i^th^ kind of inclusion, matrix and the heterogeneous material, respectively. While $\nu_{m}$ is the Poisson’s ratio of the matrix, while $c_{i}$ and $c\left( { = \mathop \sum \limits_{i}^{n} c_{i} } \right)$ is the volume concentration of the i^th^ kind of inclusion and volume concentration of the inclusion, respectively.

Also, the simplified formula for an effective shear modulus (G*) is given by^83^:

14 $$\frac{{G^{*} }}{{G_{m} }} = 1 + 15\left( {1 - \nu_{m} } \right)\mathop \sum \limits_{i = 1}^{n} \frac{{\left( {\frac{{G_{p}^{i} }}{{G_{m} }} - 1} \right)c_{i} }}{{7 - 5\nu_{m} + 2\left( {4 - 5\nu_{m} } \right)\frac{{G_{p}^{i} }}{{G_{m} }} - 2\left( {4 - 5\nu_{m} } \right)\left( {\frac{{G_{p}^{i} }}{{G_{m} }} - 1} \right)c}}$$

where, $G_{p}^{i} ,$ $G_{m} ,$ and $G^{*}$ are the bulk modulus of the i^th^ kind of particle, matrix and the heterogeneous material, respectively.

Lastly, Mori–Tanaka^84^ found a simple but applicable and powerful method to estimate the effective properties of the composite materials containing isotropic and spherical particles embedded in the matrix material. The bulk and shear moduli of an r-phase composite material are as follows^80^:

15 $$K_{hom} = \frac{{\mathop \sum \nolimits_{r} f_{r} K_{r} \left( {1 + \alpha_{0} \left( {\frac{{K_{r} }}{{K_{0} }} - 1} \right)} \right)^{ - 1 } }}{{\mathop \sum \nolimits_{r} f_{r} \left( {1 + \alpha_{0} \left( {\frac{{K_{r} }}{{K_{0} }} - 1} \right)} \right)^{ - 1 } }}$$

16 $$G_{hom} = \frac{{\mathop \sum \nolimits_{r} f_{r} G_{r} \left( {1 + \beta_{0} \left( {\frac{{G_{r} }}{{G_{0} }} - 1} \right)} \right)^{ - 1 } }}{{\mathop \sum \nolimits_{r} f_{r} \left( {1 + \beta_{0} \left( {\frac{{G_{r} }}{{G_{0} }} - 1} \right)} \right)^{ - 1 } }}$$

where, $\alpha_{0} = \frac{{3K_{0} }}{{3K_{0 + } 4G_{0} }}$ and $\beta_{0} = \frac{{6K_{0 + } 12G_{0} }}{{15K_{0 + } 20G_{0} }}$, and the subscripts “*0*” and “*r*” correspond to the matrix material and particles, respectively. The homogenized Young’s modulus and Poisson’s ratio can be calculated from the relations $E_{hom} = \frac{{9K_{hom} G_{hom} }}{{K_{hom} + G_{hom} }}$, and $\nu_{hom} = \frac{{3K_{hom} - 2G_{hom} }}{{6K_{hom} + 2G_{hom} }}$, respectively.

### SwiftComp Homogenization

**Figure 7:**
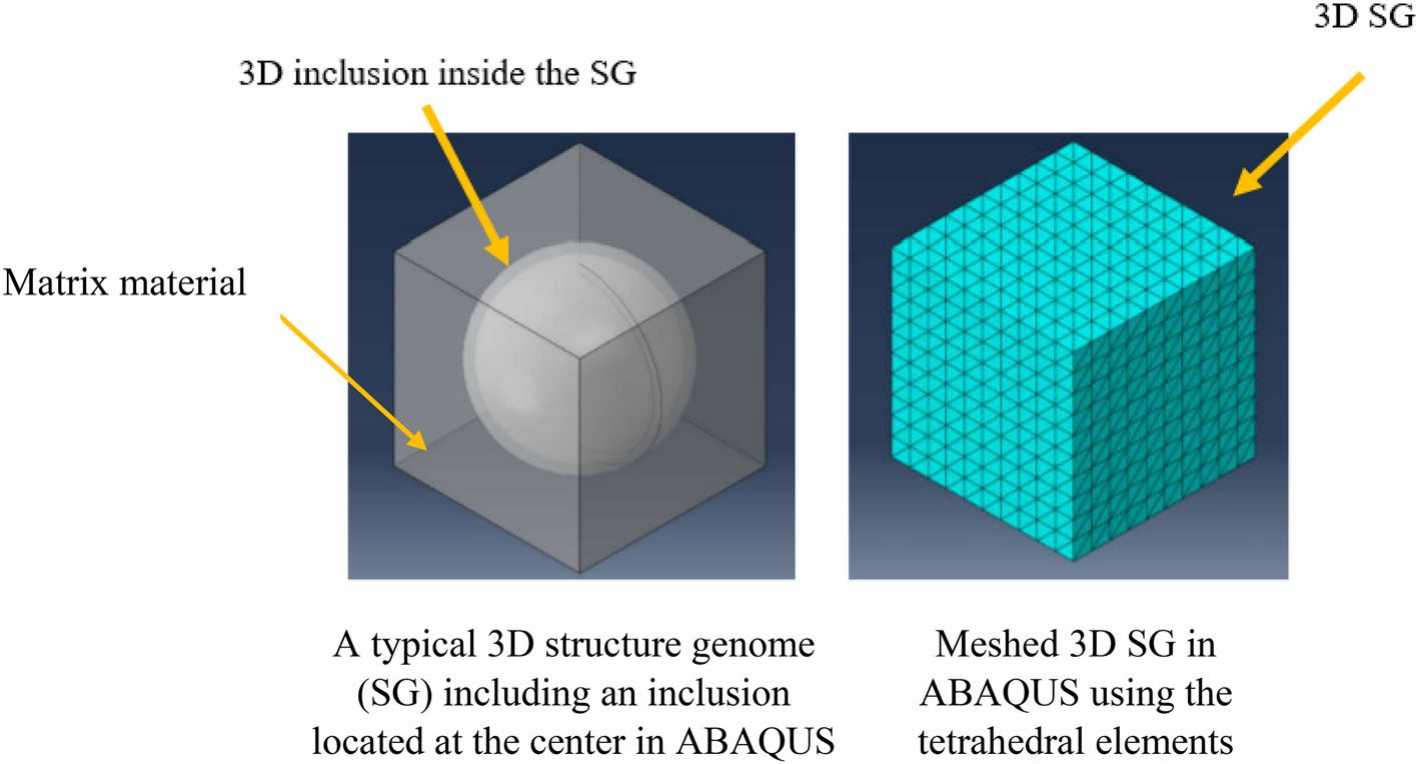
A typical 3D structure genome in SwiftComp built in ABAQUS including a 3D inclusion at the center to calculate the effective thermal and mechanical properties, also the typical meshed genome is shown in this figure^60^.

The SwiftComp can be used to homogenize the material properties using the representative volume element (RVE) analysis and in this sub-section this method is explained briefly. For this case, the software was used to obtain the compliance matrix and elastic constants of four different RVEs containing various Ni particle volume fractions analyzed, as well as the effective thermal expansion and thermal conductivities. The three-dimensional structural genomes with spherical inclusion microstructure were analyzed based on the material properties of the alloy, pores and matrix assigned in the ABAQUS model and their volume fraction properties. Figure 7 shows a typical three-dimensional (3D) structure genome (SG) with a 3D inclusion generated in ABAQUS. In this study, the RVEs contain three different phases namely the matrix, Ni particles, and porosities. For this case, a SG cannot contain two inclusions simultaneously. Therefore, the analysis was performed in two separate steps. In the first step, only the matrix and Ni particles were considered as an inclusion in the 3D SG. Then, in the second step, the effective properties of the previous step were considered as the matrix for the current 3D SG with porosities as an inclusion. The effective properties in the second 3D SG were presented as the effective properties of the RVEs.

### Fast Fourier Transform (FFT) Homogenization Technique

Fast Fourier Transform (FFT) recently has become a very popular technique to calculate the effective properties of the composites and is explained briefly. Since their inception^65,66^, computational methods based on the FFT are powerful alternatives to the classical finite-element based strategies. In their original form, the FFT-based techniques exploited the equivalence of the balance of linear momentum on the volume element under consideration and the Lippmann–Schwinger equation^85,86^, which is an integral equation for the strain field involving the strain version of the Green’s operator^87^. As the latter operator may be expressed explicitly in Fourier space, Moulinec-Suquet^65,66^ proposed to solve the Lippmann–Schwinger equation numerically by an iterative scheme, the so-called basic scheme. The geometry is discretized on a regular grid, i.e., in terms of identical brick-shaped elements, which makes this approach ideally suited for digital-volume images. Moreover, the periodic boundary conditions for the displacement field are handled naturally within this framework. The FFT-based computational homogenization methods are rather fast, as a consequence of the efficiency of the FFT implementations^67^ which require little memory, as the basic scheme is completely matrix free and operates on the strain field in place. Thus, for instance, a geometry with 512^3 voxels can be treated with only 6 GB of memory. Over the years, the FFT-based computational techniques were improved in terms of solution techniques and discretization. Moreover, the range of applicability was significantly extended. The interested readers should refer to the recent review article^88^ for a more in-depth discussion. In the current work, we used the discretization on a staggered grid^89^, as it is rather robust for microstructures with pores^90^, combined to the conjugate gradient method^91–93^ for linear elasticity, and the original Moulinec-Suquet discretization and the conjugate gradient method for thermal conductivity (see Dorn-Schneider^94^ for further details). Please note that FFT-based solvers are also available for finite element discretization on regular grids^95,96^ and boundary conditions, different from the periodic conditions^97^. We used the finite-difference discretization and periodic boundary conditions for the FFT simulations, as the latter gave rise to smaller representative volume-elements^98^.

## Results and Discussion

The predicted effective thermal and mechanical properties using various methods are presented and the results are compared to one another. The effective Young’s modulus, thermal expansion coefficient, and thermal conductivity are compared with the experimental methods and the results are compared and discussed in detail.

### Effect of Computational Homogenization Methods on Effective Mechanical Properties

**Figure 8:**
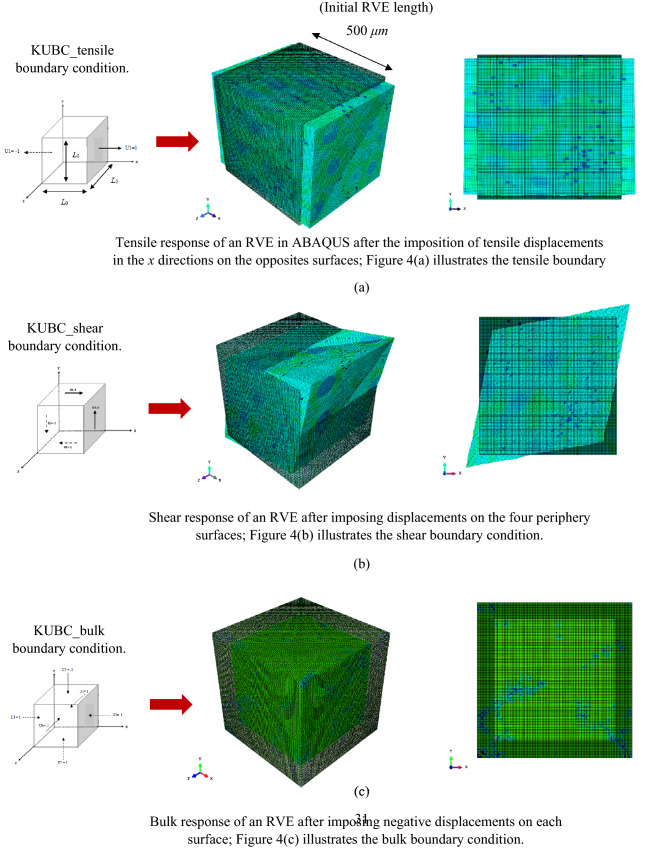
Isometric and in-plane deformed shapes of a typical RVE after (**a**) tensile loading deformation by imposing displacements at the two opposite surfaces; (**b**) shear deformation by applying shear displacement at the four periphery surfaces; and (**c**) bulk deformation by imposing negative displacement at every surface of an RVE in ABAQUS using the KUBC. The KUBC boundary conditions are explained in Fig. 4. The boundary conditions are applied on the RVEs, then the strain energies given by ABAQUS are inserted into Eq. (5) to calculate the effective moduli of the RVEs.

The effective thermo-mechanical properties of the Al_2_O_3_ material containing Ni particles and porosities were calculated numerically and compared with the experiments and this sub-section compares and analyzes the results from various methods. Figure 8 shows the deformed shape of the RVEs in ABAQUS by applying the KUBCs for the tensile, shear and bulk deformations.

Firstly, the isotropy of the generated RVEs, the RVE length and appropriate boundary conditions are investigated. The Young’s and shear moduli of the RVE containing 10% Ni and 0.9% porosity are computed for both KUBC and PBC, as presented in Table 3. The three Young’s moduli in every normal direction show little difference and the results of the three shear moduli are nearly identical. This result shows the isotropy of the RVE for the Al_2_O_3_ material containing the Ni particles and porosities. Also, as observed, the results for KUBC and PBC are very close to each other. In this study, the KUBC is used for further calculation and analysis, since the PBC is time consuming and involves considerable computational efforts in ABAQUS.

**Table 3:** Young’s and shear moduli predicted using KUBC and PBC.

Effective young’s and shear moduli (GPa)	E_1_	E_2_	E_3_	G_12_	G_23_	G_13_
Kinematic uniform boundary condition (KUBC)	306.59	306.60	306.75	124.15	124.20	124.18
Periodic boundary condition (PBC)	306.23	306.25	306.38	123.86	123.92	123.89

Furthermore, in order to verify the orthotropy, the compliance and stiffness matrices for the RVE containing 10% Ni and 0.9% calculated by the FFT-based computational homogenization technique are shown below. As observed again, the RVE is isotropic.

$$\left[ S \right] = \left[ {\begin{array}{*{20}l} {0.0033} & { - 0.0008} & { - 0.0008} & 0 & 0 & 0 \\ { - 0.0008} & {0.0033} & { - 0.0008} & 0 & 0 & 0 \\ { - 0.0008} & {0.0008} & {0.0033} & 0 & 0 & 0 \\ 0 & 0 & 0 & {0.0081} & 0 & 0 \\ 0 & 0 & 0 & 0 & {0.0081} & 0 \\ 0 & 0 & 0 & 0 & 0 & {0.0081} \\ \end{array} } \right]$$

$$\left[ C \right] = \left[ {\begin{array}{*{20}l} {351.9851} & {105.1522} & {105.1546} & {0.1679} & {0.8468} & {0.8896} \\ {105.1522} & {51.9818} & {105.0935} & {0.9265} & {0.2256} & {0.8802} \\ {105.1546} & {105.0935} & {352.0425} & {0.8907} & {0.8485} & {0.1498} \\ {0.1679} & {0.9265} & {0.8907} & {122.9425} & { - 0.4104} & { - 0.4388} \\ {0.8468} & {0.2256} & {0.8485} & { - 0.4104} & {122.9540} & { - 0.4162} \\ {0.8896} & {0.8802} & {0.1498} & { - 0.4388} & { - 0.4162} & {122.9301} \\ \end{array} } \right]$$

**Figure 9:**
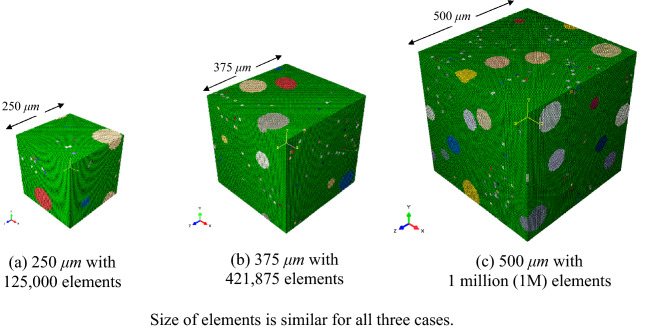
RVEs with various lengths (**a**) 250 μm; (**b**) 375 μm; and (**c**) 500 μm are created to understand the effect of the RVE size on the predicted mechanical and thermal properties.

It is important to consider an appropriate size of the RVE in the calculation of the effective properties. The RVE size must be large enough to contain sufficient features of the real material and it must be relatively small enough to keep the computational effort minimal. Therefore, the RVE size is analyzed and studied. The RVEs with lengths of 250, 375, and 500 μm are generated (Fig. 9) and their Young’s modulus is calculated, and the results are compared and shown in Table 4. The RVEs contain 10% Ni and 0.9% porosity, and it was found that the results are nearly similar. It must be emphasized that all three RVEs have identical volume fractions of the Ni particles and porosities, which is the main reason for the similar effective properties. However, it is likely that an RVE with lengths lower than 250 μm cannot keep the volume fractions constant. Thus, in this case a higher variance of results for the effective properties is expected. The 500 μm RVEs is considered for further computations in this study to provide accurate and precise results.

**Table 4:** Young’s modulus predicted using KUBC for 10% Ni and 0.9% porosity RVE with various sizes.

RVE length (μm)	250	375	500
Young’s modulus (GPa)	304.75	307.43	306.75

**Figure 10:**
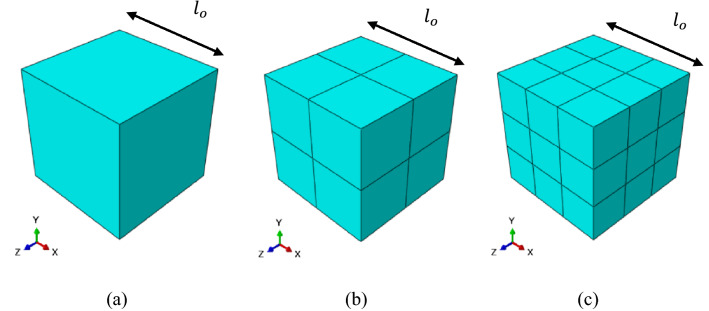
A single element (**a**) subdivision into eight elements (resolution = 1 × 2) (**b**) and 27 elements (resolution = 1 × 3) (**c**).

The effect of mesh sensitivity was studied and an RVE with initial length 500 μm containing 42,875 elements (35 elements in every direction). Every element of a RVE in ABAQUS software is subdivided into eight elements (resolution = 1 × 2) and 27 elements (resolution = 1 × 3) without changing the volume of the primary element. Figure 10 shows the element subdivision for a single element in the ABAQUS software. The element volume remains unchanged during the element subdivision. The material properties of a single element remain unchanged as well. The Young’s modulus for these three RVEs were predicted using KUBC with 10% Ni and 0.9% porosity and with initial length 500 μm. The results are presented in Table 5 and it is clearly observed that the results are not mesh sensitive. Generally, results related to the elastic and plastic region are not quite sensitive to the mesh density and mesh sensitivity is significant in fracture analysis. This matter has been shown in^99^. Furthermore, a comprehensive mesh study in RVEs containing matrix and fibers to predict the effective properties has been performed by Liu et al.^100^. It has been shown in^100^ that the effective properties converge with increasing the number of elements in an RVE.

**Table 5:** Young’s modulus predicted using KUBC for 10% Ni and 0.9% porosity RVE with length 500 μm and various element sizes.

Resolution	1 × 1	1 × 2	1 × 3
Young’s modulus (GPa)	306.94	306.78	306.72

For the upper bound, the effective Young’s, shear and bulk moduli of the RVE containing 10% Ni and 0.9% porosity are obtained and the results are presented in Table 6. The various theoretical bounds on the elastic moduli based on rule of mixtures are compared and shown in Table 6 as well. For the sake of comparison, the effective material properties computed using the SwiftComp software and FFT-based computational homogenization method for the RVEs containing 10% Ni and 0.9% porosity are added to Table 6.

**Table 6:** Comparison of theoretical bounds on homogenized elastic moduli for the RVE with 10% Ni and 0.9% porosity.

	Voigt (V)	Reuss (R)	Hill	Hashin	Mori–Tanaka	Dream.3D_ABAQUS_KUBC	Dream.3D_ABAQUS_PBC	Dream.3D_FFT	SwiftComp	Experiment
K (GPa)	192.19	3.27E-02	96.11	192.17	188.842	189.025	–	187.095	–	–
G (GPa)	127.46	2.18E-02	63.74	128.81	123.885	124.148	123.86	122.942	125.284	–
E (GPa)	312.51	6.53E-02	156.29	315.85	304.968	306.75	306.23	302.556	308.950	306.5898

The differences between the Voigt and Reuss estimates are large when the phase moduli differ by more than a factor of two, producing poor estimates in the case of the particulate composites. The large variation shown in Table 6 is consistent with the significant presence of porosity which has effectively zero Young’s modulus. It is also observed that the Hashin estimates in this case are close to the Voigt upper bound. It must be emphasized that such a discrepancy was previously observed for a typical RVE containing various phases^45^. Among these analytical methods, Mori–Tanaka predicts that the effective properties are close to the experimental results, as well as those predicted by ABAQUS by applying the KUBC and PBC. Voigt^81^ and Hashin^15^ stated that the elastic properties are higher than those estimated by the microstructural based homogenization using the SwiftComp, FFT and ABAQUS in this study. Notably, the results from the latter techniques are somewhat closer to each other. It should be noted that the FFT-based computational method predicts a relatively lower value for the moduli values compared to those predicted by the ABAQUS and SwiftComp^95^. The following relationship was obtained for the calculated moduli values using various methods:

17 $$E_{Reuss} < E_{Hill} < E_{SUBC} < E_{FFT} \approx E_{Mori - Tanaka} \approx E_{experiment} \approx \approx E_{PBC} \approx E_{{KUBC\left( {\nu \ne 0} \right)}} \approx E_{SwiftComp} < E_{{KUBC\left( {\nu = 0} \right)}} < E_{Voigt} \approx < E_{Hashin}$$

It should be noted that the values for the SUBC and KUBC with the effective Poisson’s ratio of zero are not shown in Table 6, but they have been tested to verify the equation above. Such phenomena were also reported previously^45–47^. The periphery surfaces of an RVE were constrained to create the zero effective Poisson’s ratio while applying the uniaxial tensile displacements on other surfaces.

Although, damage growth in ceramic^101^ and ductile^102,103^ material affects the properties, the problem dealt with in the current work is within an elastic regime. It means that the void nucleation in the interfacial zone between matrix and particles does not affect Young’s modulus initially during the elastic analysis. As void nucleation and growth merely happen during plastification as reported by Babout et al.^104,105^. The calculated numerical results without considering the interfacial zone were compared well with experiments in this study.

### Effective thermo-mechanical properties of RVEs

**Figure 11:**
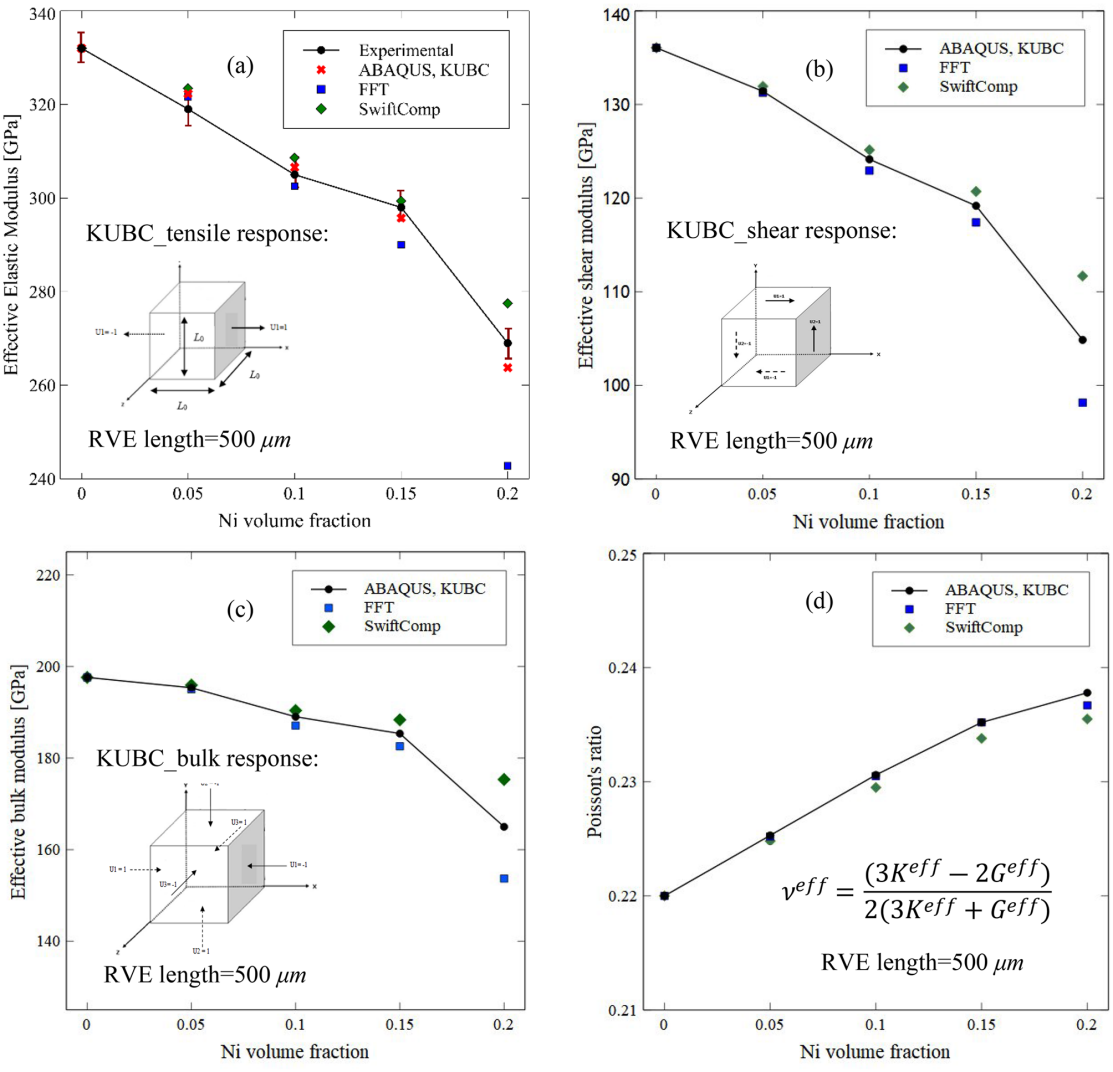
Measured and predicted effective (**a**) Young’s modulus, (**b**) shear modulus, (**c**) bulk modulus, and (**d**) Poisson’s ratio of the Al_2_O_3_-Ni composites as a function of Ni content using the KUBC in ABAQUS by applying appropriate boundary conditions and compared with various methods such as FFT and SwiftComp.

**Figure 12:**
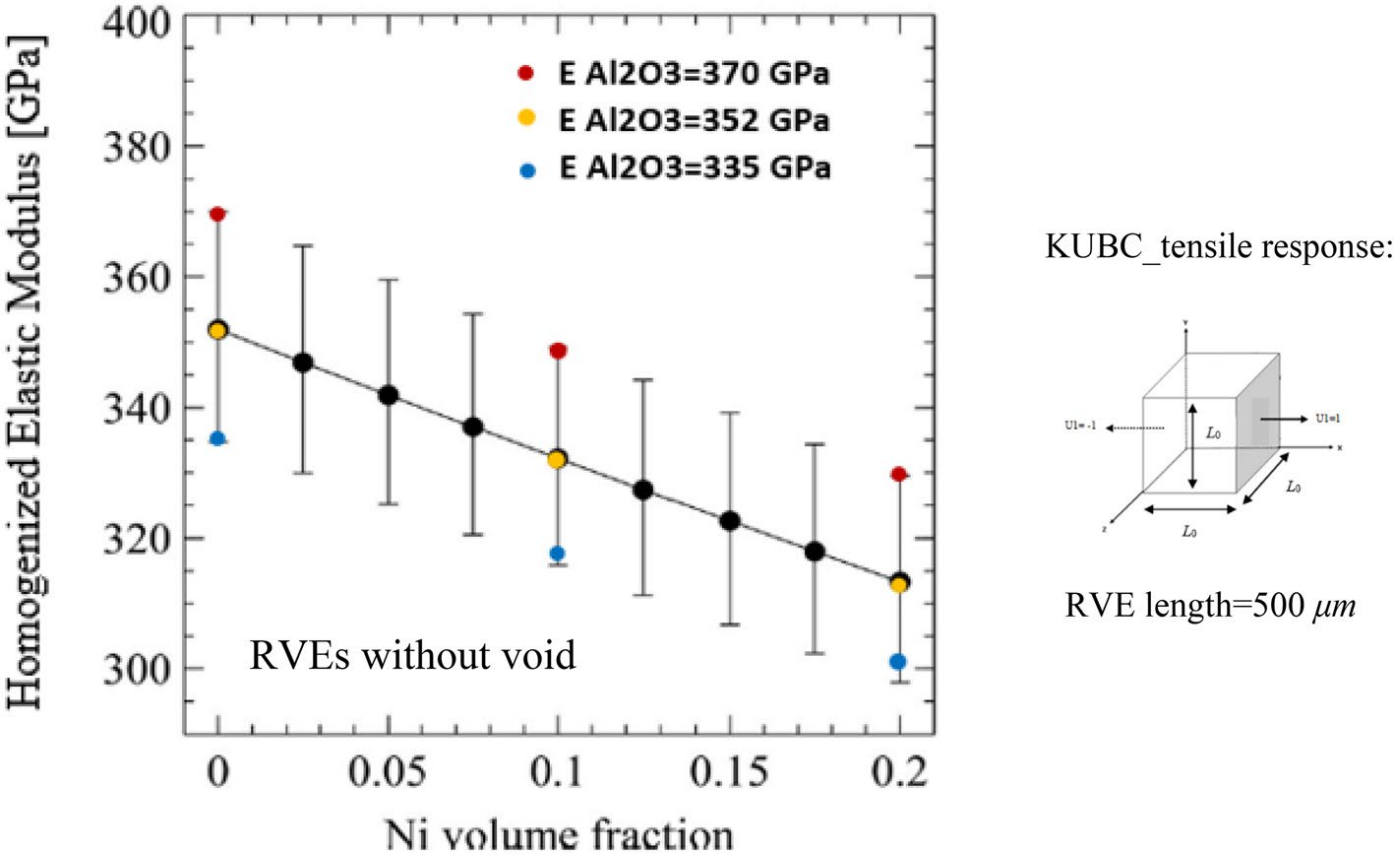
Comparison of effective Young’s modulus calculation between the computational homogenization (RVEs) and the mean-field homogenization model^70^; the black solid line corresponds to the results for 2.5% porosity while the upper and lower bounds correspond to the 0% and 5% porosity predicted in^70^, the colored dots correspond to the present RVE predictions. In this analysis, the physical porosities are absent in the RVE, but the effect of porosities has been considered on the values of the Young’s modulus of the matrix material. The values of Young’s modulus decease with the increase in the volume fraction of the porosities.

**Figure 13:**
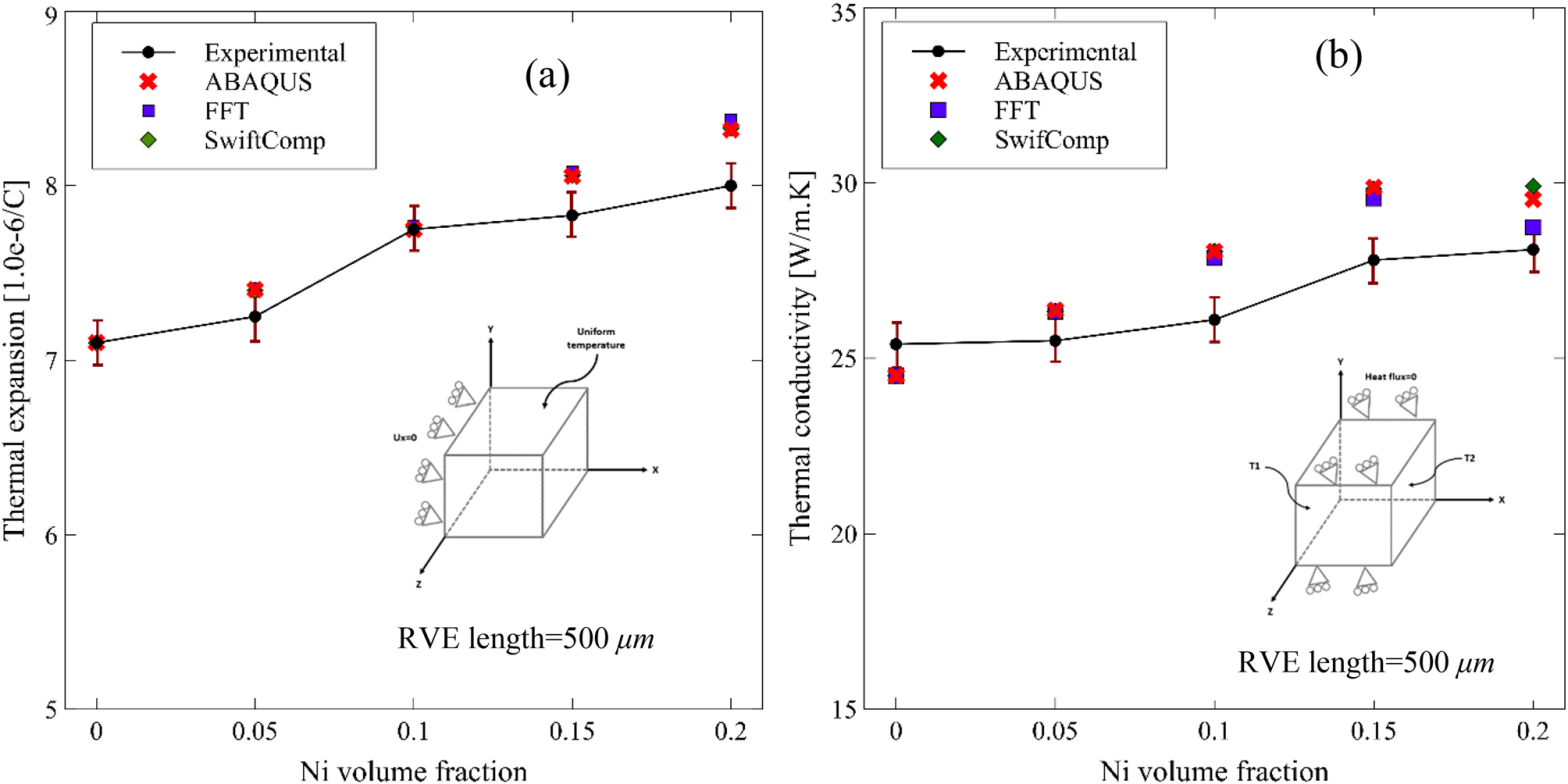
Measured and predicted (**a**) effective thermal expansion, and (**b**) effective thermal conductivity of Al_2_O_3_-Ni composites as a function of Ni content using ABAQUS by applying appropriate boundary conditions and compared using various methods such as, FFT and SwiftComp.

**Figure 14:**
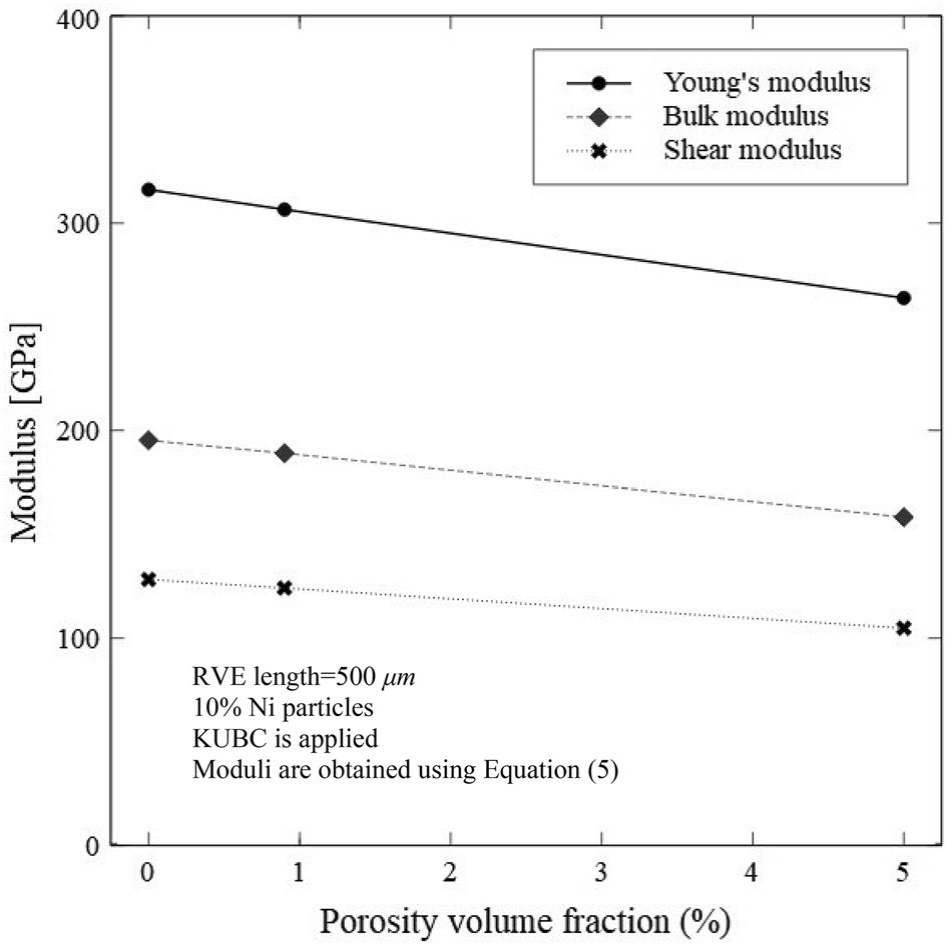
The predicted effects of porosity on the Young’s, shear and bulk moduli for 10% Ni RVE are shown here when the porosity has a significant effect on predicted effective mechanical properties by imposing KUBC in ABAQUS.

The effective thermo-mechanical properties of the RVEs containing 0, 5, 15, and 20% Ni particles with porosities (porosity volume percentages are presented in Table 2) are computed numerically using ABAQUS and the results are analyzed. The results are shown in Figs. 11, 12, 13 and 14. The effective Young’s, shear and bulk moduli of the RVEs are shown in Fig. 11a–c and the results compare well with the experimental data. The average particle size is in the range of 90 µm ~ to 100 µm and they are roughly equiaxed. On the other hand, the possible minimum volume fraction represented in Dream.3D reflects the porosities. As expected, the moduli values decrease with the increase in the Ni particle’s volume fraction since the Ni particle has lower Young’s modulus compared to the Al_2_O_3_ matrix material. Additionally, the results are compared with those presented using the SwiftComp and FFT methods. Based on the mesh convergence study in SwiftComp, we fixed the mesh size factor as 0.5 for all the RVEs. The model was then homogenized as a solid model, while the compliance matrix and the elastic constants were calculated. Since all the components of the composite were isotropic, the final composite was also isotropic. The predicted values for the Poisson’s ratio are shown in Fig. 11d. As expected, the Poisson’s ratio increases with increase of the Ni particle volume fraction since the Ni particles have a higher Poisson’s ratio than the matrix material. The Poisson’s ratio values are provided directly by the SwiftComp and FFT methods, while they are calculated using the elastic relations between the shear and bulk moduli from the ABAQUS utilizing the KUBC $\left( {\nu = \frac{3K - 2G}{{2\left( {3K + G} \right)}}} \right)$.

It is observed that the SwiftComp predicts the highest values for the Young’s, shear and Bulk moduli, while the FFT predicts the lowest value. However, it can be observed that the FFT and SwiftComp nearly predict similar values for the Poisson’s ratio as predicted by ABAQUS utilizing the KUBC.

As mentioned in “Generation of representative volume elements” and shown in Fig. 3, porosities are included in the RVE and a very small value such as 0.001 is considered for the Young’s modulus, as ABAQUS cannot take zero value for the Young’s modulus. However, as shown in a previous work^70^, a numerical model which is based on an effective medium approximation and mean-field homogenization technique is used to predict the Young’s modulus of the Al_2_O_3_ material containing various volume fractions of the Ni particles and porosities. The results of RVE calculations based on computational homogenization are compared with those presented previously^70^ where the Ni particles were present in the RVE but with the absence of the porosities. In this way, the Young’s modulus of the Al_2_O_3_ matrix material containing 0, 2.5, and 5% volume fractions are 370, 352, and 335 GPa, respectively. The Young’s modulus for the RVEs containing 0, 10, and 20% Ni particles are calculated and shown in Fig. 12. As observed, the results are in close agreement with each other.

The effective thermal expansion coefficient and thermal conductivity of RVEs were calculated using ABAQUS by imposing the boundary conditions explained in “Microstructure based homogenization using ABAQUS” (Fig. 6) shown in Fig. 13a,b, respectively. The numerical results are in close agreement with the experiments, and it is observed that the thermal expansion coefficient and thermal conductivity increase with the increase in the Ni particle volume fraction. This is expected since the Ni particles have a higher intrinsic thermal expansion coefficient and a higher thermal conductivity than the Al_2_O_3_ matrix material. The predicted effective thermal expansion and thermal conductivity coefficients were computed using the FFT-based homogenization technique and compared with those calculated by ABAQUS. As observed in Figs. 13a,b, the predicted results computed by ABAQUS, SwiftComp and FFT almost coincide. We wish to stress that the computed thermal expansion and thermal conductivity coefficients are identical at three different orientations using the SwiftComp and FFT. These results also confirm the isotropy of the RVEs.

### Effect of porosity on mechanical and thermal properties

Porosity has a significant effect on the effective material properties and an analysis is presented in this section, the effect of porosity on the mechanical and thermal properties are predicted by the RVE in ABAQUS by applying appropriate boundary conditions. The significant effect of porosity on the physical properties of sintered Al_2_O_3_ has been reported by Wang et al.^106^. As shown previously in “Effective thermo-mechanical properties of RVEs”, the effect of Ni particle volume fractions on the Young’s modulus with varying values of matrix Young’s modulus was studied and the results are shown in Fig. 12. The physical porosities were not represented in the RVE but the effect of porosities was represented through the Young’s modulus of the matrix. The ABAQUS results compare well with those obtained using the mean-field homogenization model. However, in this section, the effect of porosity on the mechanical properties of the Al_2_O_3_ composite materials are investigated and shown in Fig. 14. In fact, the volume fractions of the porosities within the RVE generated by Dream.3D are not exactly the same as inserted into this software. A small discrepancy is present between the inserted value and the present porosities volume fractions. The values of porosities volume fractions presented in Table 2 are relatively low and this volume fraction discrepancy is almost insignificant. However, the effect of porosity on the effective thermal and mechanical properties are investigated and presented in this section. Three RVEs containing 10% Ni are generated when the porosity volume is 0.0, 0.9 and 5%. As expected, the Young’s, shear and bulk moduli decrease with the increase in the volume fraction of porosity. The RVEs softens with the increase in the porosity volume fraction, and this leads to decreasing stiffness. Also, as the porosity possesses the lowest value for the Young’s modulus which is almost zero, we deduce from the rule of mixture that the increase in the porosity volume fraction lowers the effective mechanical properties.

**Figure 15:**
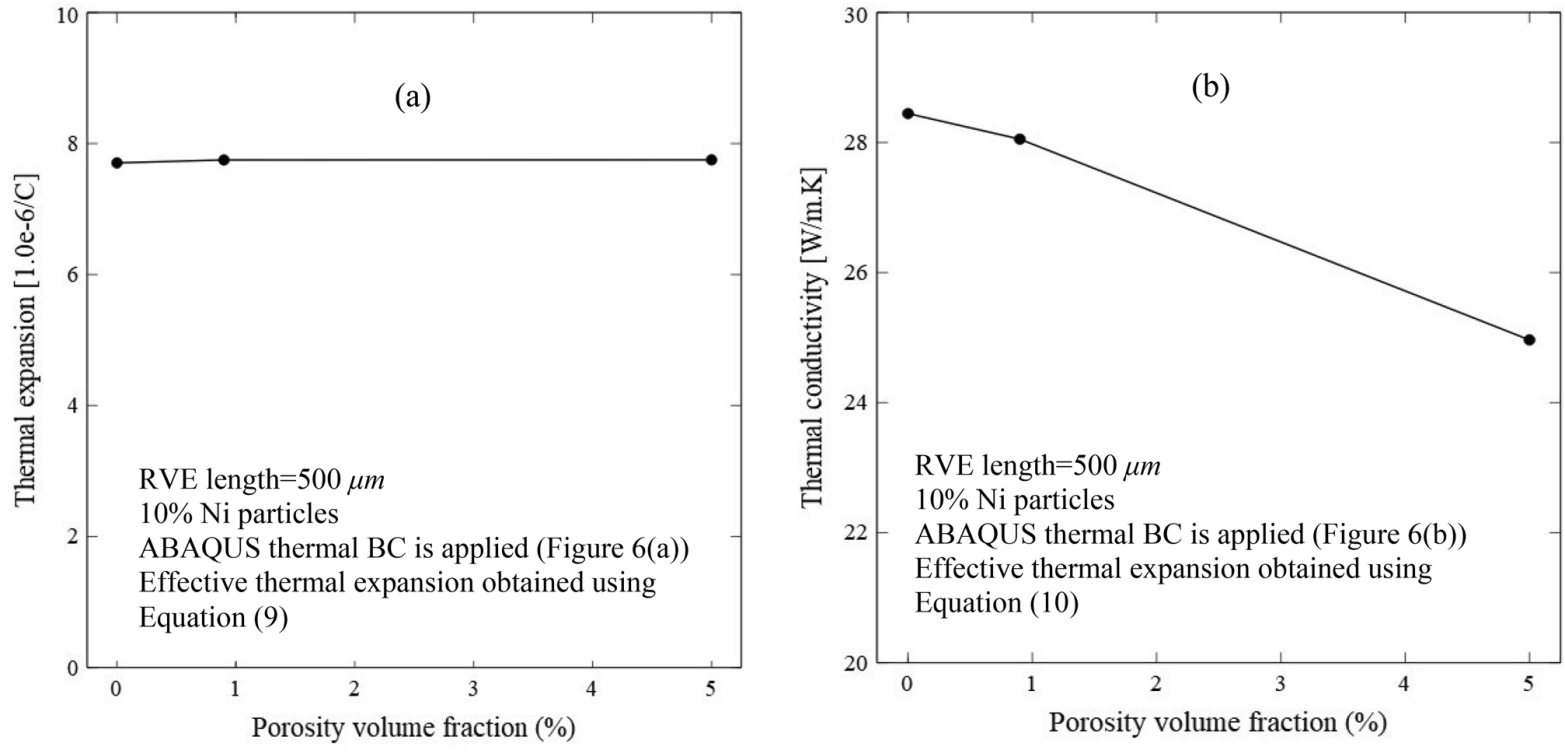
The predicted effect of porosity on (**a**) effective thermal expansion, and (**b**) effective thermal conductivity for 10% Ni RVE.

The effect of porosity on the thermal expansion is studied and the results are shown in Fig. 15a. As observed, the porosity has an insignificant effect on the thermal expansion. As studied by Ghabezloo^107^, there is an absence of a unique and significant thermal expansion-porosity volume fraction relationship for all porous metals. A parametric study was performed and the effective thermal expansion of the RVEs with various constituents, thermal and mechanical properties was computed. The increase or decrease in the thermal expansion depends on the combination of a set of parameters including the Young’s modulus and thermal expansion of matrix and particles. The effective thermal expansion increases when one of the solid constituents has higher thermal expansion but lower rigidity compared to the other constituents^107^. However, in the present RVE, only one constituent (Ni particles) is present. Thus, the results show negligible and insignificant thermal expansion coefficients with changes in the porosity volume fraction. Furthermore, this effect could be deduced from the Turner model to calculate the effective thermal expansion coefficient^108^.

18 $$\alpha^{eff} = \frac{{\alpha_{m} E_{m} f_{m} + \alpha_{p} E_{p} f_{p} }}{{E_{m} f_{{m + E_{p} f_{p} }} }}$$

where, $\alpha , E$ and $f$ are thermal expansion coefficient, Young’s modulus and volume fractions, respectively. Here, *eff*, *m*, and *p* denote the effective, matrix and constituents’ values, respectively. Only $f_{m}$ decreases with the porosity as a constituent and affects both the nominator and denominator in Eq. (18). So, it possesses an insignificant effect on the effective thermal expansion coefficient.

The effect of porosity on the thermal conductivity was studied and the results are shown in Fig. 15b. As observed, the porosity has a significant effect which lowers the thermal conductivity as the content increases. In constant, the porosity has an insignificant effect on the thermal expansion. The decrease in the thermal conductivity with the increase in the porosity is associated with a higher thermal resistance due to the gas-phase reduction conduction at higher levels of porosity. Additionally, the decrease in the effective thermal conductivity with the increase of void volume fraction can be explained by a rule of mixture namely the Maxwell–Eucken 1 (ME1) as follows^109^:

19 $${K}^{eff}=\frac{{K}_{m}{f}_{m}+{K}_{p}{f}_{p}\frac{3{K}_{m}}{2{K}_{m}+{K}_{p}}}{{f}_{m+{f}_{p}}\frac{3{K}_{m}}{2{K}_{m}+{K}_{p}}}$$

where $K$ is the thermal conductivity coefficient. Also, *eff*, *m*, and *p* denote the effective, matrix and constituents’ values, respectively. The thermal conductivity of porosities is zero and the matrix volume fraction decreases with the increase in the porosity volume fraction which leads to a decrease in the $K^{eff}$ as shown in Eq. (19).

## Conclusions

The effective properties of the RVEs for Ni-reinforced Al_2_O_3_ composites in various compositions for designing cutting inserts were computed by computational homogenization applied to RVEs using the finite element method. The effective thermal and mechanical properties such as the thermal expansion coefficient and thermal conductivity, as well as the Young’s, shear and bulk moduli are predicted and compared with the experiments. The results presented in this study clearly show that the mesoscale effective properties can be predicted using a micro-mechanics RVE based approach, as the results are in fair comparison with the experiments.

Firstly, an RVE study was performed to investigate the material orthotropy, RVE size selection, and the effect of ABAQUS boundary conditions as well as various other techniques on effective properties. The orthotropy of the material was tested and it was found that the RVEs are isotropic as the moduli are similar in all directions. The results obtained using KUBC and PBC are similar as well and the results are presented using KUBC to shorten the computational time. The mechanical properties were predicted using the SwiftComp and they closely match with those predicted by KUBC. Additionally, the mechanical properties were predicted using the FFT-based computational homogenization technique and it is observed that they are slightly lower than those predicted by KUBC. The mechanical properties are also compared with the rules of mixture, and it was found that the values of the upper bound are higher than those predicted by experiments and other computational methods. The Poisson’s ratio values of the RVEs are presented by the SwiftComp, FFT and ABAQUS and the values are almost identical.

Then, the effect of Ni particle and porosity volume fractions on the effective material properties were examined. The Ni particles have lower Young’s modulus and higher thermal conductivity and expansion coefficients compared to the Al_2_O_3_ matrix material. The effective thermal and mechanical properties of the RVEs decreases and increases, respectively, with the increase in the Ni particle volume fraction. The effect of porosity on the thermal and mechanical properties of the RVEs was studied and it is observed that the mechanical Young’s, shear and bulk moduli decrease with the increase in the porosity volume fraction. On the contrary, the porosity has an insignificant effect on the effective thermal expansion.

Overall, a comprehensive ABAQUS result comparisons with experiments and other computational methods were performed and it can be concluded that the RVE generated by Dream.3D imported into ABAQUS is capable of further analyses using the finite element method. The proposed computational design methodologies, which have been validated by experimental data as a result of a novel synthesis route, are expected to aid researchers and the cutting tool industry in developing new inserts with tailored properties.

## Data Availability

The datasets generated and analyzed during the current study are available from the corresponding author on reasonable request.

## Abbreviations

*A*_*p*_: Projected contact area measurement of the Young’s modulus in indentation test
b: Compliance constant measurement of the Young’s modulus in indentation test
${\mathrm{c}}_{\mathrm{i}}$: Volume concentration of inclusions
${C}_{ij} (i=\mathrm{1,2},..,6)$: Stiffness matrix components
$dL$: Length change in an RVE
$$dT$$: Applied temperature
*E*_*i*_: Young’s modulus of diamond indenter
${E}_{i} (i={1,2},..,6)$: Edges in an RVE
${E}_{IT}$: Young’s modulus of sample
${E}_{ij}, {G}_{ij},\,{\rm and}\,K$: Young’s, shear, and bulk moduli
${{E}_{hom}, G}_{hom}\,{\rm and}\,{K}_{hom} (i={1,2},3)$: Effective Young’s, shear and bulk moduli in Mori–Tanaka rule of mixture.
*E*_*r*_: Reduced modulus
${f}_{i}$: Volume fraction of particles in the rule of mixtures
${F}_{i} (i={1,2},..,6)$: Faces in an RVE
${G}_{p}^{i}$, ${G}_{m}$, and $\,{G}^{*}$: Shear moduli of the ith kind of inclusion, matrix and the heterogeneous material, respectively in Hashin’s rule of Mixture.
*h*_*c*_: Contact depth measurement of the Young’s modulus in indentation test
*L*_0_: Initial size of a cubical RVE
${L}_{x},{L}_{y},\,{\rm and}\,{L}_{z}$: Initial length of RVEs in x, y and z directions
$k$: Thermal conductivity coefficient
${K}_{p}^{i}$, ${K}_{m}$, and ${K}^{*}$: Bulk moduli of the ith kind of inclusion, matrix and the heterogeneous material, respectively in Hashin’s rules of mixture.
K* and G*: Effective bulk and shear moduli in the rule of the mixture-based Voigt upper bound.
$$q$$: Heat flux
*n*: Number of inclusions in the rules of mixtures
$\%P$: Percentage of porosity
$S$: Slope of the unloading curve measurement of the Young’s modulus in indentation test
${S}_{ij} (i={1,2},..,6)$: Compliance matrix components
x, y, and z: Cartesian coordinates
$$U$$: Strain energy
${V}_{RVE}$: Volume of an RVE
${v}_{i} (i={1,2},..,8)$: Vertices in an RVE
$\alpha$: Thermal expansion coefficient
${\gamma }_{ij} (i, j={1,2},3)$: Engineering shear strain components
$${\delta }_{ij} (i, j={1,2},3)$$: Kronecker delta
$${\varepsilon }_{ij} (i, j={1,2},3)$$: Strain components
$${\varepsilon }_{ij}^{avg}$$: Average strain
$\mu\,{\rm and}\,\sigma$: Particle size distribution parameters in Dream.3d
*ν*_*i*_: Poisson’s ratio of diamond indenter
${\nu }_{ij} (i, j={1,2},3)$: Poisson’s ratio of components
${\nu }_{m}$: Poisson’s ratio of matrix
*ν*_*s*_: Poisson’s ratio of samples
${v}_{v}$: Volume fraction of voids
$$\rho$$: Density
$${\sigma }_{ij} (i, j={1,2},3)$$: Stress components
*ϕ*: Volume fraction
